# Europium(iii)/terbium(iii) mixed metal–organic frameworks and their application as ratiometric thermometers with tuneable sensitivity in organic dispersion†

**DOI:** 10.1039/d5ra00822k

**Published:** 2025-04-09

**Authors:** Madhura Joshi, Maurizio Riesner, Zhuang Wang, Sahba Mireskandari, Raju Nanda, Rebecca Elfriede Reber, Christoph Huber, Marcus Fischer, Rachel Fainblat, Karl Mandel, Dorothea Wisser, Doris Segets, Gerd Bacher, Florian M. Wisser, Martin Hartmann

**Affiliations:** a Erlangen Center for Interface Research and Catalysis (ECRC), Friedrich-Alexander-Universität Erlangen-Nürnberg (FAU) Egerlandstraße 3 91058 Erlangen Germany florian.wisser@fau.de martin.hartmann@fau.de; b Werkstoffe der Elektrotechnik, University of Duisburg-Essen (UDE) Carl-Benz-Straße 199 47057 Duisburg Germany gerd.bacher@uni-due.de; c Institute for Energy and Materials Processes – Particle Science and Technology (EMPI-PST), University of Duisburg-Essen (UDE) Carl-Benz-Straße 199 47057 Duisburg Germany doris.segets@uni-due.de; d Department of Chemistry and Pharmacy, Inorganic Chemistry, Friedrich-Alexander Universität Erlangen-Nürnberg (FAU) Egerlandstrasse 1 91058 Erlangen Germany; e Fraunhofer-Institute for Silicate Research ISC Neunerplatz 2 97082 Würzburg Germany; f Center for Nanointegration Duisburg-Essen (CENIDE), University of Duisburg-Essen (UDE) Germany; g Erlangen Center for Functional Particle Systems (FPS), Friedrich-Alexander-Universität Erlangen-Nürnberg (FAU) Haberstr. 9a 91058 Erlangen Germany

## Abstract

The ability to engineer on a molecular level luminescent metal–organic frameworks (MOFs) enables the design of well-performing ratiometric, *i.e.*, self-referencing, temperature sensors. Lanthanide-based MOFs stand out as luminescent temperature sensors due to their high luminescence intensity and the sharp emission lines of the lanthanides. The use of two different lanthanide cations, incorporated into the same MOF structure, is supposed to enable ratiometric temperature sensing. Herein, we present a series of mixed-metal Eu_*x*_Tb_(1−*x*)_BTC, in which the metal ions are homogeneously dispersed, as demonstrated by ^1^H solid state NMR spectroscopy. The Eu_*x*_Tb_(1−*x*)_BTC series shows controllable luminescent properties, which depend on the solvation of the lanthanide. The two MOFs in the series with the lowest Eu contents, namely Eu_0.05_Tb_0.95_BTC and Eu_0.02_Tb_0.98_BTC, are suitable candidates for ratiometric temperature sensing, achieving sensitivities of up to 2.0% K^−1^. As the fluorescence is affected by the presence of solvents, simultaneous ratiometric temperature and solvent sensing is possible with remarkable high thermal sensitivities of *ca.* 0.1% K^−1^ and *ca.* 0.2% K^−1^ for dispersions of Eu_0.02_Tb_0.98_BTC in acetonitrile and ethanol, respectively.

## Abstract 2

Unprecedented insight into the lanthanide dispersion in mixed-metal Eu_*x*_Tb_1−*x*_BTC using high-field ^1^H MAS NMR spectroscopy. Isolation of Eu sites in the TbBTC matrix leads to efficient Tb-to-Eu energy transfer and high temperature sensitivity near RT.

## Graphical abstract

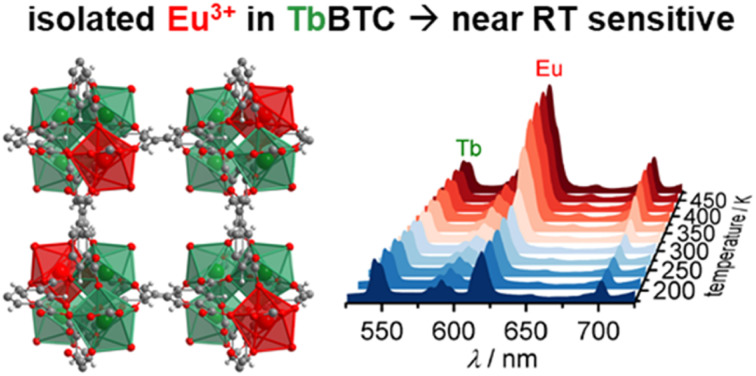

## Introduction

In the field of chemical sensing, metal–organic frameworks (MOFs) have attracted much attention as they combine the advantages of high surface area and pore volume as well as crystallinity with tuneable structures and functionalities.^1,2^ Thus, MOFs have found applications in easy-to-read optical sensors,^3–5^ as well as in electrochemical sensing^6,7^ and fluorescent sensing.^2,8–12^ MOF-based fluorescence sensors stand out compared to other materials due to their modular synthesis, allowing adjustment of the MOF structure and chemical composition and, thus, tailoring of its optical properties. This has in the past allowed the design of fluorescence sensors with high target selectivity as well as high sensitivities and low limits of detection.^12^ MOF-based fluorescence sensors have found applications in various fields, including toxic metal ion detections in wastewater, gas and volatile organic compounds detection, but also for determination of physical properties such as pH, humidity and temperature.^2,8–17^

To enable ratiometric sensing, *i.e.* self-calibrating sensing without the use of an external reference, at least two different luminescent centres are required.^10^ This can include two different metal complexes, *e.g.* two different lanthanides, two different types of quantum dots (QDs), or a combination of a QD and a metal complex. Lanthanide-based (Ln) MOFs are attracting increasing attention due to their good luminescence intensity and the sharp emission lines of the lanthanides.^8,18^ Thus, from two or more transitions, originating from the different emissive centres within the same material, the sensor signal is determined optically as the ratio between the probe-dependent emission intensities. In case of temperature sensing, the nature of the MOF, *i.e.*, the combination of metal and organic linker, mainly determines the temperature range which can be accurately measured. MOFs as ratiometric temperature sensors might offer large potential as an *in situ* probe to detect local temperature changes during a given application, *e.g.* in biological applications and nanomedicine, in catalytic reactions or in adsorption processes.^16^ However, precise understanding of the temperature sensing under different conditions is required. Though it is known that the luminescence in lanthanide 1,3,5-benzene tricarboxylate (LnBTC) MOFs strongly depends on the experimental conditions, *e.g.* the presence of a solvent,^19–21^ systematic studies that allow understanding the interplay between the MOF material and its functional environment are yet rare.^21^

With regard to particle size and shape, most of the known pure Ln-containing MOFs show a preferred crystallization along one crystallographic direction leading typically to micrometer-sized, needle-shaped crystals.^22–24^ While this is very attractive for crystal structure determination, it may hamper their application in areas where size becomes important, such as in contrasting agents in bio-imaging, or in thin films. A prominent technique to control the size and shape of MOF crystals is the use of modulators. In their seminal work, Guo *et al.* reported the use of sodium acetate (NaOAc) as a suitable capping agent to reduce the particle size without affecting the crystal structure and properties of LnBTC MOFs.^25^ While the original protocol allowed for the synthesis of rod-like LnBTC of several tens of μm in length,^8,25^ the protocol reported by Guo *et al.*^25^ lead to the synthesis of almost spherical particles with diameters below 100 nm. Those LnBTC (nano)particles have found widespread application in luminescent sensing, including small molecule detection,^20,26^ and ratiometric temperature sensing,^27^ or in luminescent thin films.^25,28–30^ However, detailed understanding of Ln incorporation in mixed LnBTCs, and the impact of solvation on ratiometric temperature sensing remain yet unsolved.

Here we report the investigation of the ratiometric temperature sensing in a series of mixed europium and terbium BTC MOFs, labelled as Eu_*x*_Tb_(1−*x*)_BTC. The homogeneous incorporation of both cations was demonstrated by ^1^H MAS NMR spectroscopy, as the ^1^H chemical shift of the linker is sensitive to the presence of both lanthanides. Using the NaOAc modulator approach, the Eu_*x*_Tb_(1−*x*)_BTC series was obtained as nanoparticulate systems (nanorods), enabling their use not only in the dry solid state, but also dispersed in various solvents in the colloidal state. Temperature sensing was established on dry powders of Eu_0.05_Tb_0.95_BTC and Eu_0.02_Tb_0.98_BTC. Finally, we demonstrate the potential of dual emissive Eu/Tb MOFs towards simultaneous ratiometric temperature and solvent sensing. To this end, we have studied the temperature-dependent luminescence of Eu_0.02_Tb_0.98_BTC dispersed in ethanol or acetonitrile as model organic solvents. The emission intensities and the ratio between the Eu and Tb emission depend on both the temperature and the solvation of the MOF, thus allowing simultaneous temperature and solvent determination.

## Results and discussion

### Mixed-metal Eu/Tb BTC MOFs

**Fig. 1:**
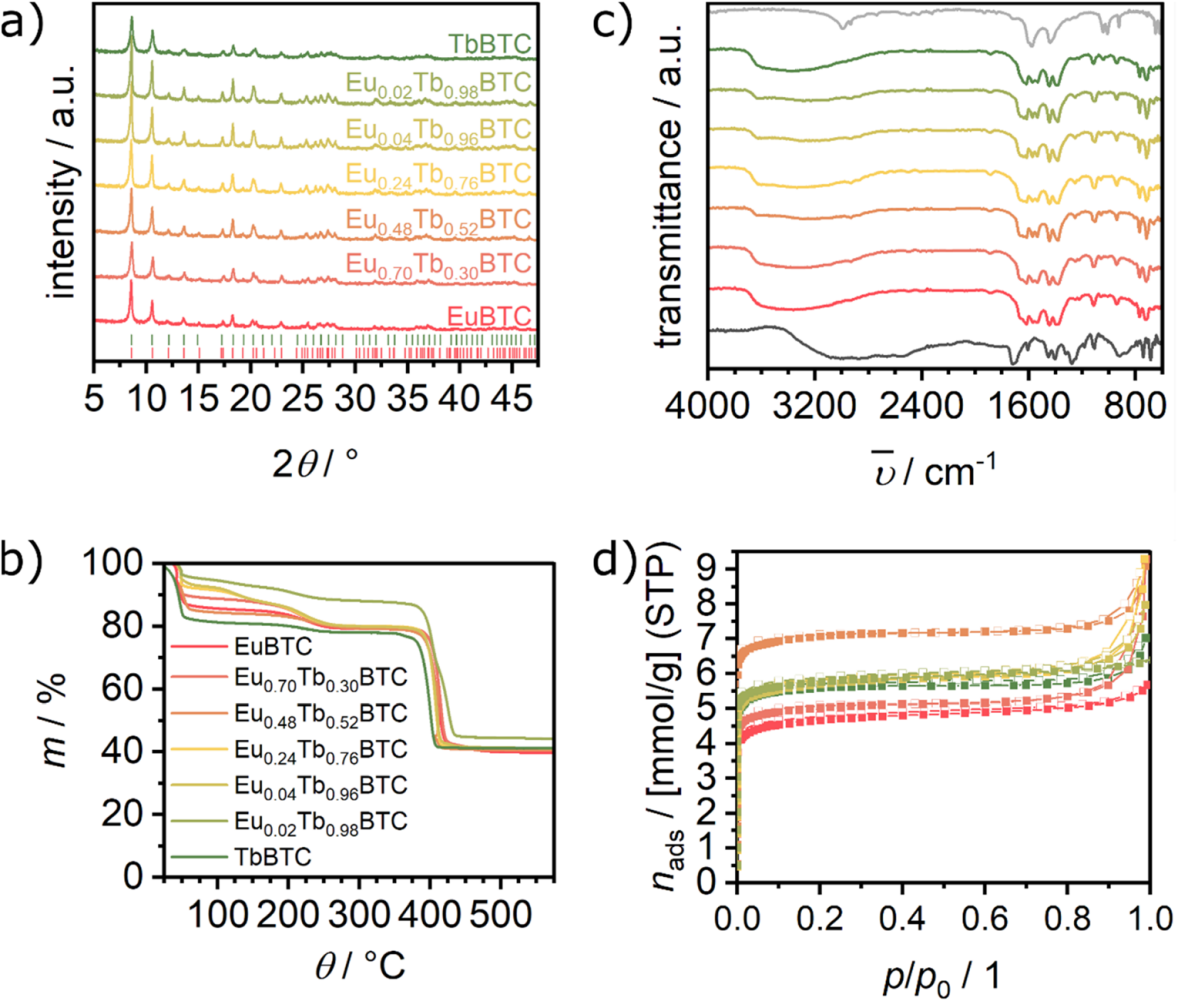
(a) PXRD patterns of as-synthesised Eu_*x*_Tb_(1−*x*)_BTC MOFs. Bragg marker for [EuBTC (H_2_O)]·(H_2_O)_1.5_ (red, CCDC 617492 (ref. 19)) and [TbBTC(H_2_O)]·(DMF)(H_2_O)_0.5_ (green^31^). (b) TGA recorded under synthetic air (2 K min^−1^ heating rate), (c) IR spectra of Eu_*x*_Tb_(1−*x*)_BTC MOFs (same colour code as in (a)) together with the IR spectrum of H_3_BTC (dark grey) and NaOAc (light grey) and (d) N_2_ physisorption isotherms recorded at 77 K (same colour code as in (a)). Closed symbols denote the adsorption, open symbols indicate the desorption branch.

The PXRD patterns of the as-synthesised Eu_*x*_Tb_(1−*x*)_BTC MOFs (*x* = 0.0 to 1.0) are very similar and show the characteristic peaks of the parent EuBTC^19^ and [TbBTC(H_2_O)]·(DMF)(H_2_O_0.5_) (Fig. 1a and S1†).^31^ The high similarity of the patterns confirms that a series of isostructural MOFs was obtained. As the materials were obtained as fine powders (Fig. S2†), they exhibit rather broad peaks. Thus, small changes in lattice parameters cannot be observed, which might be expected when gradually replacing Eu with Tb. In the series the estimated crystallite sizes, as obtained using the Scherrer equation, vary between 28 (TbBTC) and 46 nm (Eu_0.02_Tb_0.98_BTC, see Table S1†). All materials in the Eu_*x*_Tb_(1−*x*)_BTC series were obtained as needle-shaped nanorods (Fig. S3†). For the two most interesting materials, Eu_0.05_Tb_0.95_BTC and Eu_0.02_Tb_0.98_BTC (see below), the characteristic length of the particles is below 500 nm, with average lengths of 125 and 225 nm, respectively (Fig. S2 and S3†).

The thermal stability and decomposition behaviour of all materials is very similar, as observed by thermogravimetric analysis (TGA) in air. Between room temperature and 275 °C, desorption of remaining ethanol, water and/or DMF occur, while network decomposition only starts above 350 °C. In line with the expected values for the two pure phases, the relative mass loss above 350 °C changes from approx. 49% in case of EuBTC to approx. 47% in case of TbBTC. At 600 °C Eu_2_O_3_ and Tb_4_O_7_ are obtained,^32^ at ∼1000 °C, Tb_4_O_7_ is deoxygenised yielding Tb_2_O_3_ (Fig. S4†).^32,33^ The results from TGA, combined with results from inductively coupled plasma optical emission spectroscopy (ICP OES), are thus indicative for an almost perfect 1 : 1 stoichiometry between lanthanides and BTC. Indeed, using ICP OES, only traces of sodium (<1%, Table 1) are detected.

**Table 1:** Overview of metal content (*ω*_i_), apparent surface area estimated using the BET theory (*S*_BET_) and total pore volume (*V*_p_) in the series of LnBTCs

Material	*ω*_Eu_ a/wt%	*ω*_Tb_ a/wt%	*ω*_Na_ a/wt%	*S*_BET_ b/m^2^ g^−1^	*V*_p_ c/cm^3^ g^−1^
EuBTC	31.88 ± 0.15	—	0.49 ± 0.01	410	0.24
Eu_0.75_Tb_0.25_BTC	23.09 ± 0.61	8.16 ± 0.05	0.60 ± 0.02	450	0.32
Eu_0.51_Tb_0.49_BTC	15.84 ± 0.91	15.81 ± 0.11	0.39 ± 0.11	630	0.31
Eu_0.25_Tb_0.75_BTC	7.79 ± 0.30	23.87 ± 0.19	0.22 ± 0.04	510	0.32
Eu_0.05_Tb_0.95_BTC	1.47 ± 0.08	33.41 ± 2.85	0.46 ± 0.07	500	0.28
Eu_0.02_Tb_0.98_BTC	0.62 ± 0.04	34.41 ± 0.66	0.38 ± 0.06	510	0.22
TbBTC	—	31.77 ± 0.24	0.18 ± 0.03	510	0.20

a Determined by ICP OES analysis.

b Determined from N_2_ physisorption experiments at 77 K, at 0.05 < *p*/*p*_0_.

c Determined from N_2_ physisorption experiments at 77 K, at 0.99 *p*/*p*_0_.

The almost perfect stoichiometry is further evidenced by IR spectroscopy. The vanishing of the characteristic *υ*_as_(COO) vibration of H_3_BTC around 1720 cm^−1^ in all MOFs of the series highlights that hardly any missing cluster defect is present (Fig. 1c). Such a defect would give rise to only partially deprotonated H_3_BTC, which would in turn be characterised by its vibration around 1720 cm^−1^. Likewise, only traces of acetate might be present in this series of samples, as the characteristic CH vibrations around 2990 and 2930 cm^−1^ are barely visible. The apparent surface area of the series was evaluated using nitrogen as probe molecule. After activation at 250 °C in vacuum for at least 4 hours, all MOFs show type I isotherms. The BET areas vary between 410 and 630 m^2^ g^−1^ for EuBTC and Eu_0.48_Tb_0.52_BTC, respectively (Table 1). All MOFs show a pore volume of approx. 0.2 to 0.3 cm^3^ g^−1^ (Table 1). The BET areas of the MOFs are in line with literature reports on Tb and Eu MOFs,^26,31^ but are slightly lower as compared to their Ho, Tm, Lu and Dy counterparts.^34^

### ^1^H solid state NMR spectroscopy

**Fig. 2:**
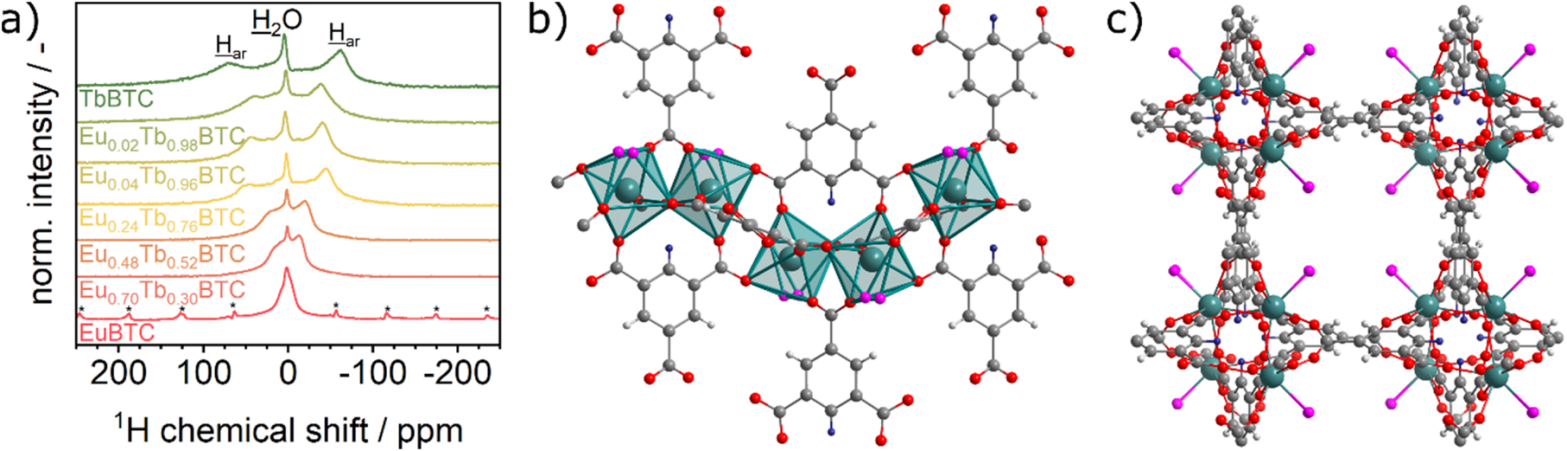
(a) ^1^H MAS NMR spectra of Eu_*x*_Tb_(1−*x*)_BTC recorded at 500 MHz. Asterisks denote spinning sidebands. To all spectra a line broadening of 300 Hz was applied. (b and c) View of the crystal structure of [TbBTC(H_2_O)]·(DMF)(H_2_O)_0.5_ (ref. 31) along the crystallographic a direction (b) and along the crystallographic *c* direction (c). Solvent molecules in the pore channel are omitted for clarity. Colour code: green: Tb, grey: C, white: H (crystallographic H1 position), blue: H (crystallographic H2 position), red: O (from BTC) and pink: O (from water, disordered between two positions).

Recently, Blahut *et al.* demonstrated the use of magic angle spinning (MAS) nuclear magnetic resonance (NMR) spectroscopy as a powerful toolbox to obtain insight into fine details of the structure of paramagnetic MOFs.^35–37^ Here, we recorded ^1^H MAS NMR spectra at fast MAS of 30 kHz on the single lanthanide MOFs, EuBTC and TbBTC, and the mixed-metal phases Eu_*x*_Tb_(1−*x*)_BTC (Fig. 2a). Both single-metal MOFs are paramagnetic, however, Eu^3+^ in the ground state of the [Xe] 4f^6^ configuration often exhibits an angular momentum of 0, leading to no paramagnetic shift anisotropy, contact shift or pseudo-contact shift, whereas Tb^3+^ exhibits sizable values for these paramagnet-induced interactions.^38^ This is also reflected by different aspects of the ^1^H MAS NMR spectra: the ^1^H MAS NMR spectrum of pure EuBTC shows three resonances and well-defined spinning sidebands (Fig. 2a). We tentatively assign the resonance at approx. 1.6 ppm to the protons of the water molecule coordinated to the lanthanides in the MOF structure and the resonances at *ca.* 8 and −4 ppm to the two different proton positions of the aromatic protons of BTC present in the asymmetric unit of both EuBTC and TbBTC (Fig. 2b and c).^19,31^ For comparison, in the ^1^H NMR spectrum of paramagnetic Cu_3_BTC_2_, only one resonance for aromatic protons has been observed,^39,40^ since the asymmetric unit of Cu_3_BTC_2_ only contains one proton position.^41^

The ^1^H MAS NMR spectrum of pure TbBTC contains three resonances. The resonance of the water molecules is shifted to 4.7 ppm. The resonances of the aromatic protons are shifted to *ca.* 70 ppm and −62 ppm. These extreme values of ^1^H chemical shifts are caused by significant paramagnetic contributions, including shift anisotropy, contact shifts or pseudo-contact shifts induced by Tb^3+^. Interestingly, in the ^1^H MAS NMR spectra of the series of mixed metal Eu_*x*_Tb_(1−*x*)_BTC MOFs, with increasing Tb^3+^ content, we observe an almost continuous evolution of the chemical shifts of the two aromatic proton positions from one pure phase to the other. Thus each proton in the mixed metal Eu_*x*_Tb_(1−*x*)_BTC MOFs is influenced by paramagnetic effects of both metal ions. This confirms experimentally that the two cations are homogeneously distributed within the MOFs, leading to short Tb^3+^-to-Eu^3+^ interatomic distances (*d*_Tb–Eu_ ∼4.7 Å) and excluding the formation of large Eu- or Tb-rich domains or even co-crystallization of single metal MOFs. This unprecedented insight into the cation distribution is crucial for the understanding of other material properties including their photophysics (*vide infra*).

### Room temperature luminescent properties

**Fig. 3:**
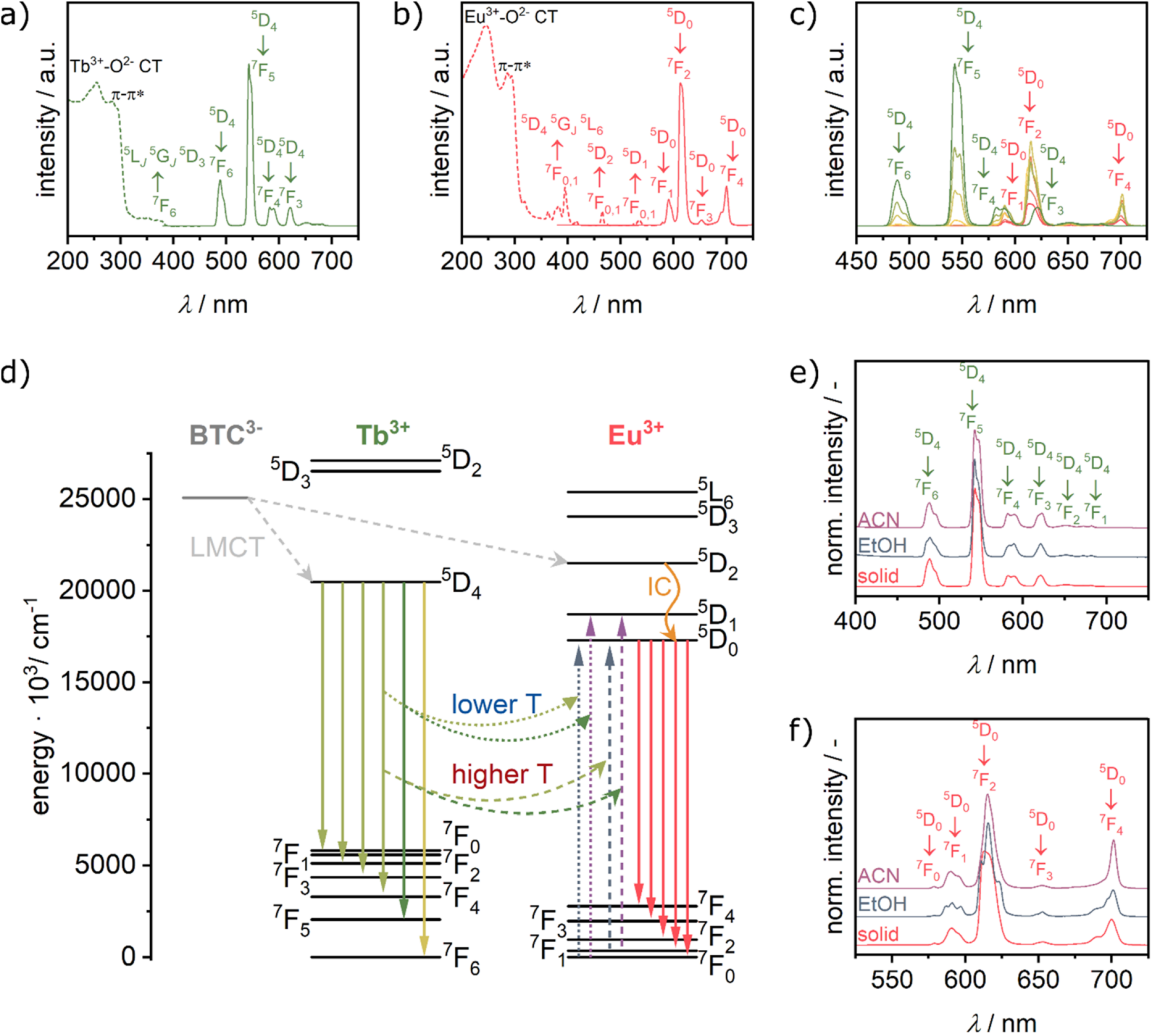
(a) PL (solid line, *λ*_ex_: 254 nm) and PL excitation (dotted line, *λ*_em_: 543 nm) spectra of TbBTC, (b) PL (solid line, *λ*_ex_: 254 nm) and PL excitation (dotted line, *λ*_em_: 615 nm) spectra of EuBTC, (c) PL spectra of Eu_*x*_Tb_(1−*x*)_BTC MOFs [*x* = 0.0 to 1.0] in the solid state (*λ*_ex_: 254 nm). Colour code as in Fig. 2a. Green labels mark Tb^3+^-based emission, red labels Eu^3+^-based emission. (d) Simplified Jablonski diagram showing basic photophysical processes according to ref. 42 and 43 LMCT: ligand-to-metal charge transfer, IC: internal conversion. Comparison of the solid-state PL spectrum with the PL spectra obtained for the MOF dispersions in ethanol and acetonitrile of (e) TbBTC and (f) EuBTC (*λ*_ex_: 300 nm).

The photoluminescence (PL) spectra of the samples were measured in the solid state and dispersed in ethanol and acetonitrile. In the solid state, upon excitation at *λ*_ex_ = 254 nm, the pure TbBTC shows the characteristic emissions centred at approx. 489 nm (^5^D_4_ → ^7^F_6_), 545 nm (^5^D_4_ → ^7^F_5_), 586 nm (^5^D_4_ → ^7^F_4_) and 622 nm (^5^D_4_ → ^7^F_3_), respectively (Fig. 3a). Likewise, the PL spectrum of EuBTC shows the lines characteristic of Eu-centred emissions at 595 nm (^5^D_0_ → ^7^F_1_), 616 nm (^5^D_0_ → ^7^F_2_), 653 nm (^5^D_0_ → ^7^F_3_) and 695 nm (^5^D_0_ → ^7^F_4_, Fig. 3b).^44^ The corresponding excitation spectra of both pure MOFs demonstrate an efficient sensitisation by the linker,^18^ as the excitation mainly occurs on ligand based π–π* transitions and Ln^3+^–O^2−^-charge transfer (CT) bands.^45^ We suppose that similarly to literature reports, ligand-to-metal charge transfer occurs from linker-based triplet states to ^5^D_4_ (Tb^3+^) and ^5^D_2_ (Eu^3+^) levels (Fig. 3d).^42^ In case of EuBTC, relaxation by internal conversion (IC) from the ^5^D_2_ into ^5^D_0_ energy level occurs. From the lowest exited levels (^5^D_0_ (Eu^3+^) or ^5^D_4_ (Tb^3+^)), transition of the electron into ^7^F_*J*_ levels is accompanied by emission of a photon.^42,46^

In the PL spectra of the Eu_*x*_Tb_(1−*x*)_BTC series, the intensities of the Tb^3+^-based emission lines decrease and vanish almost completely from an Eu content of 25% onwards (Fig. 3c and S5†). Such a decrease of the Tb^3+^ emission intensity upon doping the framework with Eu has also been observed in, *e.g.*, series of [Tb_2*x*_Eu_2−2*x*_(bdc)_3_(H_2_O)_4_]_∞_ or Eu_*x*_Tb_1−*x*_DMBDC MOFs.^47–49^ Recent work by Carneiro Neto *et al.*^50^ and Trannoy *et al.*^14^ suggest that the energy transfer occurs by a quadrupole–quadrupole mechanism, as the acceptor Eu^3+^ ions are homogeneously dispersed in the Tb^3+^ donor matrix (see above), resulting in a minimal distance between neighbouring Tb^3+^ and Eu^3+^ ions of 4.75 Å.^31^ A basic requirement for the energy transfer process among two species is the spectral overlap between donor emission (in our case Tb^3+^) and acceptor absorption (in our case Eu^3+^). This requirement is fulfilled at around 490 nm, 540 nm and 580 nm with the overlap between the ^5^D_4_ → ^7^F_6_, ^5^D_4_ → ^7^F_5_ and ^5^D_4_ → ^7^F_4_ emission of Tb^3+^ and the ^7^F_0,1_ → ^5^D_2_, the ^7^F_0,1_ → ^5^D_1_ and ^7^F_0,1_ → ^5^D_0_ absorption of Eu^3+^ (Fig. S6†).^43,50^ It is noteworthy that ^7^F_1_ → ^5^D_0_ exhibits larger oscillator strength than ^7^F_0_ → ^5^D_0_.^51^ The energy transfer becomes more efficient upon heating due to the increased number of energy transfer channels given by the thermal population of acceptor (Eu^3+^) ^7^F_1_ and ^7^F_2_ levels (Fig. 3d).^43^

The intensity of the Eu^3+^ emission lines at 595 nm (^5^D_0_ → ^7^F_1_), 616 nm (^5^D_0_ → ^7^F_2_), 653 nm (^5^D_0_ → ^7^F_3_) and 695 nm (^5^D_0_ → ^7^F_4_) increases for low Eu ratios and decreases for higher Eu ratios (Fig. 3c). The decrease in Eu^3+^ emission with increasing Eu content might be attributed to concentration quenching in form of energy migration from Eu^3+^ to a non-radiative recombination site or by cross relaxation with other (excited) Eu sites.^48^

The emission spectra of dispersions of the pure TbBTC in ethanol or in acetonitrile are very similar to the PL spectra in the solid state (Fig. 3e). The absence of any significant solvatochromic behaviour in TbBTC is corroborated by reports on molecular Tb^3+^ complexes.^52–54^ In case of EuBTC, significant solvatochromic effects are observed, in particular on the ^5^D_0_ → ^7^F_2_ (Δ*J* = 2 manifold) and ^5^D_0_ → ^7^F_4_ (Δ*J* = 4 manifold) transition (Fig. 3f). When dispersed in ethanol, the emission spectrum of EuBTC is characterised by a slight shift of the emission wavelength and a more pronounced Stark splitting of the Δ*J* = 2 manifold: instead of one broad band, three well resolved bands can be distinguished. When replacing ethanol with acetonitrile, the Stark splitting of the ^7^F_2_ manifold is less pronounced, with an almost unaffected position of the highest energy transition. These changes in Stark splitting of the manifold have been correlated to the change of the solvent polarity. With increasing solvent polarity, the difference between lower energy transitions decreases, causing a less pronounced ligand field splitting of the ^7^F_*J*_ levels.^55^ Likewise, also more pronounced Stark splittings are observed for the Δ*J* = 1 and Δ*J* = 2 manifolds. For the Δ*J* = 4 manifold, the Stark splitting is almost invisible when EuBTC is dispersed in acetonitrile, resulting in an increase in the relative intensities of the transition as compared to the PL spectra recorded in ethanol and in the dry solid state. Overall, the solvatochromic behaviour of the Eu^3+^ luminescent centres follows the same trends observed for organometallic Eu complexes.^55^ The observed changes are thus most likely caused by a change in the solvent polarity,^55^ but contributions from solvent coordination to open metal sites and/or H-bonding cannot be ruled out. The different solvatochromic behaviours of Eu^3+^ and Tb^3+^ luminescent centres in EuBTC and TbBTC are also observed in the Eu_*x*_Tb_(1−*x*)_BTC series (Fig. S7†), opening the possibility for dual ratiometric temperature and solvent sensing (*vide infra*).

### Temperature-dependent solid-state luminescence properties

**Fig. 4:**
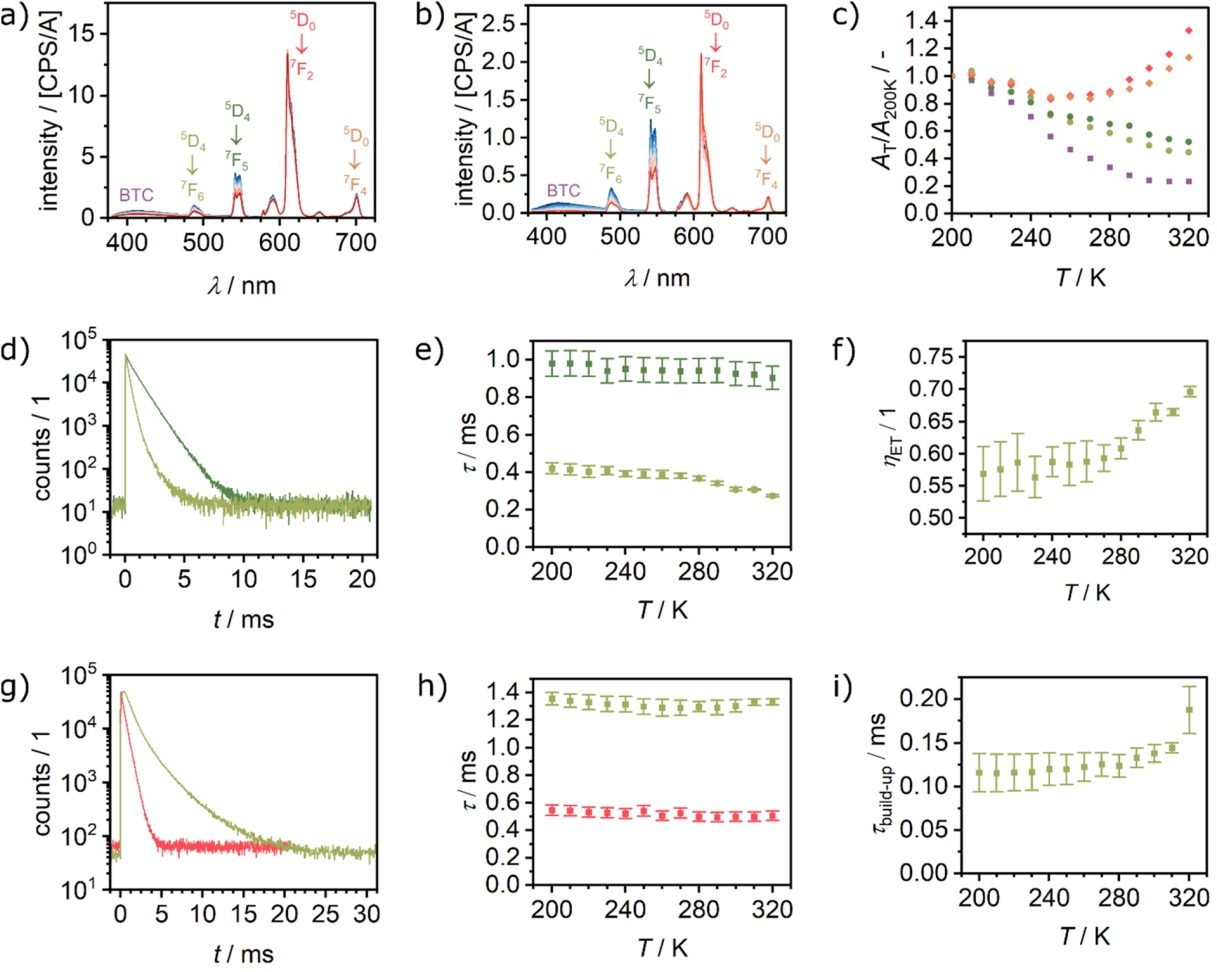
Temperature dependent luminescence spectra recorded between 200 and 330 K after excitation at 370 nm of (a) Eu_0.05_Tb_0.95_BTC and (b) Eu_0.02_Tb_0.98_BTC. (c) Corresponding evolution of the luminescence with temperature, expressed as area at a given temperature divided by the area at 200 K, for Eu_0.02_Tb_0.98_BTC. (d) Time correlated single photon counting (TCSPC) spectra of TbBTC (dark green) and Eu_0.02_Tb_0.98_BTC (light green) recorded at *λ*_em_ = 542 nm (Tb emission) and at 320 K. For all TCSPC spectra between 200 and 320 K see Fig. S12a and d.† (e) Evolution of Tb emission lifetime as a function of temperature in TbBTC (dark green) and Eu_0.02_Tb_0.98_BTC (light green). (f) Corresponding energy transfer efficiency from Tb to Eu, estimated from the Tb emission lifetimes shown in (b) using eqn (4). (g) TCSPC spectra of EuBTC (red) and Eu_0.02_Tb_0.98_BTC (light green) recorded at *λ*_em_ = 610 nm (Eu emission) and at 320 K. For all TCSPC spectra between 200 and 320 K see Fig. S12e and f.† (h) Evolution of Eu emission lifetime as a function of temperature in EuBTC (red) and Eu_0.02_Tb_0.98_BTC (light green). (i) Evolution of Eu emission build-up as a function of temperature in Eu_0.02_Tb_0.98_BTC (light green).

Temperature-dependent luminescence spectra were recorded for Eu_0.05_Tb_0.95_BTC and Eu_0.02_Tb_0.98_BTC in the temperature range from 200 to 330 K and from 173 to 423 K in the solid-state (Fig. 4a, b and S8†). The terbium-based emission, *e.g.* at 489 nm and 543 nm, decreases with increasing temperature. For the Eu^3+^-based emission, the temperature dependency is more complex. Between 200 and 280 K, the emission also decreases with increasing temperature, while above 280 K it increases again (Fig. 4c and S9†). This change in temperature dependency further confirms an energy transfer from donor Tb^3+^ levels to acceptor Eu^3+^ levels, which becomes more efficient in the Eu_*x*_Tb_(1−*x*)_BTC series above approx. 280 K. The thermal energy is then sufficient to allow for the thermal population of acceptor (Eu^3+^) ^7^F_1_ and ^7^F_2_ levels (Fig. 3d).^43^ As mentioned above, energy transfer becomes thus more efficient due to the increased number of energy transfer channels (see below). Moreover, this change in the temperature dependency of the Eu emission is not correlated to a phase transition of the MOFs or a phase separation into single metal LnBTCs, evidenced by the absence of any signal in dynamic scanning calorimetry experiments (Fig. S10†).

For both materials, in the range from 200 to 280 K, the decrease in emission intensity is stronger for the Tb^3+^ than for the Eu^3+^-based emission. For single lanthanide Ln_2_(Hpcpa)_3_(H_2_O)_5_·H_2_O (H_3_pcpa = *N*-(4-carboxyphenyl)oxamic acid) a decrease of the emission of either Tb^3+^ or Eu^3+^ with increasing temperature (15 to 300 K) has been reported.^44^ For EuBTC doped with CsPbBr_3_ quantum dots,^27^ as well as Eu_0.37_Tb_0.63_BTC,^18^ an increase in Eu^3+^ emission with increasing temperature above 293 K has been reported. In contrast, for a series of Eu_*x*_Tb_(1−*x*)_-DMBDC (*x* = 0.0011, 0.0046, 0.0069; DMBDC = 2,5-dimethoxy-1,4-benzenedicarboxylate) a different behaviour is observed.^56^ While the Tb^3+^ emission still decreases with increasing temperature, the Eu^3+^ emission increases with increasing temperature in the range from 10 to 300 K. Interestingly, for pure Eu-DMBDC and pure Tb-DMBDC, a decrease of the emission with increasing temperature has been reported, the same temperature dependency behaviour as observed for pure TbBTC and EuBTC (Fig. S11†). This inversion of the temperature dependency of the Eu^3+^ emission when doped into the Tb-DMBDC matrix has been explained by an efficient energy transfer from Tb^3+^ to Eu^3+^.^56^

To confirm the energy transfer in the Eu_*x*_Tb_1−*x*_BTC series, we analysed the emission lifetimes of the pure lanthanide as well as mixed lanthanide materials as a function of the temperature (Fig. 4d, g and S12†). The Tb emission lifetime in TbBTC decreases from approx. 980 μs at 200 K to ∼900 μs at 320 K, while in Eu_0.02_Tb_0.98_BTC it decreases from approx. 420 to 270 μs (Fig. 4e and Table S2†). From those measurements, the efficiency of the energy transfer (*η*_ET_) from Tb to Eu can be estimated by comparing the Tb lifetime in TbBTC (*τ*_0_) with that in Eu_*x*_Tb_1−*x*_BTC materials (*τ*_mi*x*_) according to^18,56^

1 *η*_ET_ = 1 − *τ*_0_/*τ*_mix_.

The efficiency of the energy transfer increases from approx. 55% (200 K) to more than 70% (320 K, Fig. 4f). The energy transfer efficiency is comparable to the best efficiencies reported for Eu–Tb MOFs with comparable composition and Tb^3+^-to-Eu^3+^ interatomic distances (*d*_Tb–Eu_ < 4.5 Å, *η*_ET_ ∼80%).^14,57^ It is much higher as compared to MOFs with larger Tb^3+^-to-Eu^3+^ interatomic distances (*d*_Tb–Eu_ > 7 Å), which cause significantly lower energy transfer efficiencies (20–40%).^58^

Interestingly, the build-up time constant of the Eu emission in Eu_0.02_Tb_0.98_BTC increases significantly compared to the pure EuBTC (Fig. 4i). The build-up also increases with increasing temperature from ∼120 μs (200 K) to ∼190 μs (320 K). Such an increase in the Eu build-up time constant has recently been attributed to a delayed Tb^3+^-to-Eu^3+^ energy transfer.^59^ Consequently, the lifetime of the Eu emission in the mixed Eu_0.02_Tb_0.98_BTC remains almost constant (∼1.2–1.3 ms), a significant increase compared to lifetimes between 550 μs (200 K) and 500 μs (320 K) in pure Eu BTC (Fig. 4h and Table S2†). Overall, these results, the shortening of the Tb lifetime, the increase in the Eu lifetime and the increase in the energy transfer efficiency, are in excellent agreement with experimental and computational models of the Tb-to-Eu energy transfer in MOFs.^14,15^

### Ratiometric temperature sensing in the solid-state

**Fig. 5:**
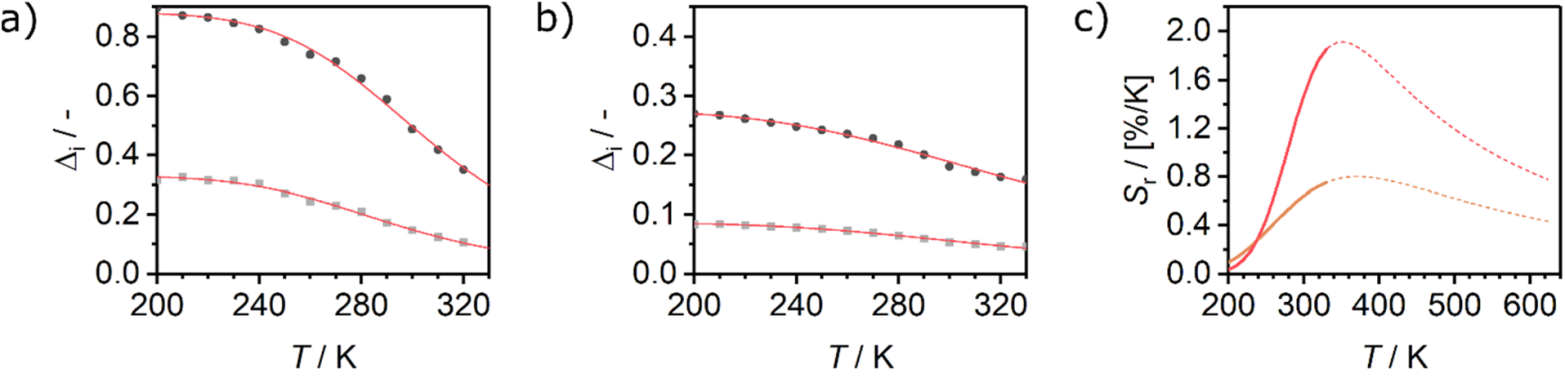
Evolution of the luminescence ratios *Δ*_*i*_ as a function of temperature, for (a) Eu_0.02_Tb_0.98_BTC and (b) Eu_0.05_Tb_0.95_BTC. Data for *Δ*_*i*_ based on Tb ^5^D_4_ → ^7^F_5_ transition are shown in dark grey, based on Tb ^5^D_4_ → ^7^F_6_ transition in light grey. (c) Evolution of relative sensitivities in the analysed temperature regime for Tb ^5^D_4_ → ^7^F_5_ transition. *S*_r_ of Eu_0.02_Tb_0.98_BTC (red), *S*_r_ of Eu_0.05_Tb_0.95_BTC (orange). Solid lines correspond to *S*_r_ curves in the calibrated temperature regime, dotted lines correspond to the extrapolated *S*_r_ curves limited by the thermal decomposition of the MOFs around 623 K (Fig. 1b).

For the application as ratiometric temperature sensor, we used the ratio2
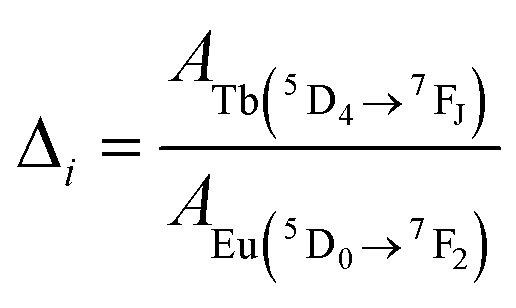
of the integrated area of the Tb^3+^ emission (^5^D_4_ → ^7^F_5_ or ^5^D_4_ → ^7^F_6_) with respect to the integrated area of the europium emission (^5^D_0_ → ^7^F_2_, Fig. 5a and b). The integrated area of the transitions originating from Tb^3+^ presents the stronger thermal quenching (Fig. 4c and S9†), most likely involving one deactivation channel, while the transitions originating from Eu^3+^ are thermally less affected. Therefore, we analysed the data using the Mott–Seitz expression for one non-radiative recombination channel:^14,16,60^3
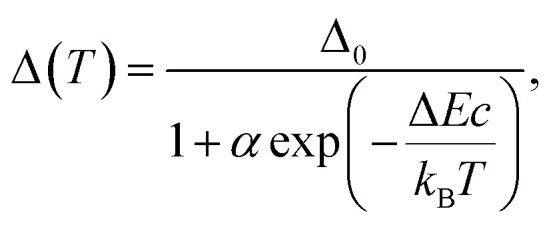
with *Δ*_0_ being the ratio *Δ* for the limiting case *T* → 0 K, *α* the ratio between non-radiative and radiative decay rate for the limiting case *T* → 0 K, Δ*E* the activation energy for the non-radiative decay rate in cm^−1^ and *c* the conversion factor from cm^−1^ into Joule in [J cm^−1^]. To evaluate the performance of the two mixed metal BTCs, we calculated the relative thermal sensitivity *S*_r_ according to:^16,60^4
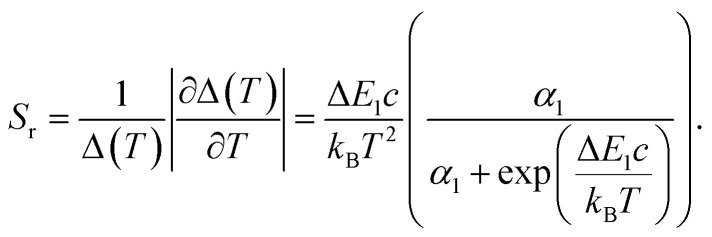
*S*_r_ is usually given in the unit of percent of change per Kelvin [% K^−1^]. Since its first description in 2003, *S*_r_ has been widely accepted as figure of merit to compare the performance of different thermometers.^16^ In the calibrated low temperature range between 200 and 330 K, maximum sensitivities *S*_m_ at 330 K of up to 1.85% K^−1^ and 0.75% K^−1^ were achieved for Eu_0.02_Tb_0.98_BTC and Eu_0.05_Tb_0.95_BTC, respectively. These values are comparable to the sensitivities reported for other mixed Tb-Eu-MOFs around 300 K (Table 2).

**Table 2:** Overview of the temperature range studied, activation energies for the non-radiative decay rates Δ*E* and maximum relative sensitivities *S*_m_ for mixed Eu–Tb MOFs

Material	*T*/K	Δ*E*/cm^−1^	*S*_m_/[% K^−1^]	*T*_m_/K	Ref.
Eu_0.05_Tb_0.95_BTC	200–330	1300 ± 100	0.75	330	
	298–473	6100	1.19	347	
Eu_0.02_Tb_0.98_BTC^a^	200–330	2600 ± 500	1.61 ± 0.24	330	
	173–473	5500 ± 400	1.66 ± 0.39	335 ± 5	
Eu_0.37_Tb_0.63_BTC	313–473		0.68	313	18
Tb_0.85_Eu_0.15_(OAc)(1,3-bdc)(H_2_O)	150–350	500	0.44	236	14
Tb_0.99_Eu_0.01_(bdc)_1.5_(H_2_O)_2_	290–320	—	0.14	318	61
Tb_0.8_Eu_0.2_BPDA	293–328	—	1.19	313	62
Eu_0.33_Tb_0.66_(ad)_0.5_(phth)(H_2_O)_2_	303–423	—	1.21	303	63
Eu_0.05_Tb_1.95_-PDC	298–333	—	1.37	333	64
[Tb_1.9_Eu_0.1_(N-BDC)_3_(DMF)_4_]	100–293	918	2.6	190	15
Tb_5.94_Eu_0.06_-UiO-66	255–295	—	4.9	295	65
Fe_3_O_4_@ZrOBDC:Eu^3+^,Tb^3+^	153–573	—	0.7	543	66

a Average of two independent experiments. 1,3-bdc = 1,3-benzendicarboxylate; bdc = 1,4-benzenedicarboxylate; BPDA = biphenyl-3,5-dicarboxylate; ad = adipate; phth = phthalate; PDC = pyridine-3,5-dicarboxylate; N-BDC = 2-amino-1,4-benzenedicarboxylate.

When extrapolating the sensitivity *S*_r_ range up to the thermal stability limit of the MOFs around 623 K, we estimate the overall sensitivities to increase to *ca.* 2.1% K^−1^ (∼370 K) and *ca.* 0.8% K^−1^ (∼370 K) for Eu_0.02_Tb_0.98_BTC and Eu_0.05_Tb_0.95_BTC, respectively. Indeed, in the calibrated high temperature range between 298 and 473 K, maximum sensitivities *S*_m_ of up to 2.0% K^−1^ (335 K) and 1.19% K^−1^ (347 K) were achieved for Eu_0.02_Tb_0.98_BTC and Eu_0.05_Tb_0.95_BTC, respectively (Table 2). Note that the average *S*_m_ and *T*_m_ values obtained for Eu_0.02_Tb_0.98_BTC using two different setups and on independent samples are statistically the same. Moreover, the materials show no change in structural properties (Fig. S13 and S14a†) and only slight changes in photophysical properties (Fig. S14b and c†).

Interestingly for macroscopic Eu_0.37_Tb_0.63_BTC needles, preferred oriented along the crystallographic *c*-direction,^18^ the *S*_r_ value decreases above 313 K with increasing temperature. To shed light on the origin of the observed different luminescent properties of macroscopic needles (Eu_0.37_Tb_0.63_BTC) reported by Wang *et al.*^18^ and our materials, we analysed the emission lifetimes of the pure lanthanide as well as mixed lanthanide materials as a function of the temperature (Fig. 4d, g and S12†). The emission lifetime of both Eu and Tb behave quite differently in the pure LnBTC materials of different morphology: While we observe lifetimes of around 900 μs and 500 μs for Tb and Eu respectively at 320 K (Fig. 4e and h), Wang *et al.* observed much longer lifetimes of approx. 1300 μs for Tb (313–330 K).^18^ In Eu_*x*_Tb_1−*x*_BTC materials, the Tb emission lifetime is always shorter than in the pure TbBTC, with however shorter lifetimes in the case of the smaller particles (∼300 μs *vs.* ∼550 μs at 310 K).

Consequently, the more pronounced relative reduction of the Tb emission lifetime in Eu_*x*_Tb_1−*x*_BTC compared to TbBTC for the nanoscopic needles results in a higher energy transfer efficiency of approx. 70% at 320 K, compared to ∼58% at 313 K for macroscopic needles.

While the lifetime of the Eu emission in Eu_0.02_Tb_0.98_BTC shows hardly any temperature dependency (∼1.2–1.3 ms, Fig. 4h), Wang *et al.* observed an increase in lifetime for Eu in the mixed material as well. A possible reason for these differences in behaviour might be a smaller crystallite size in the case of our materials, different preferred orientation, different amount and nature of defects present in the materials (*e.g.* NaOAc *vs.* HCl used as modulators), or the different excitation wavelength used (385/370 nm *vs.* 296 nm). The excitation wavelength affects the ratios of Tb and Eu emission (Fig. S15†) and thus possibly the energy transfer. The use of different modulators might cause different coordination polyhedra and site symmetry in the materials, parameters which are known to impact the luminescence lifetime of *e.g.* Eu.^67^

Like in the Tb_1−*x*_Eu_*x*_(OAc)(1,3-bdc)(H_2_O) series reported by Trannoy *et al.*,^14^ we see a slightly higher maximum temperature for lower europium content. However, in the case of BTC frameworks, the highest sensitivity is also observed for the lowest europium content. A possible explanation might be that the temperature dependencies of the Tb centred transitions are less pronounced in Eu_0.02_Tb_0.98_BTC than in Eu_0.05_Tb_0.95_BTC (Fig. 4c and S9†). This is also seen by the increase in activation energy for the non-radiative decay with decreasing Eu content (Table 2).

### Ratiometric temperature sensing in organic solvents

**Fig. 6:**
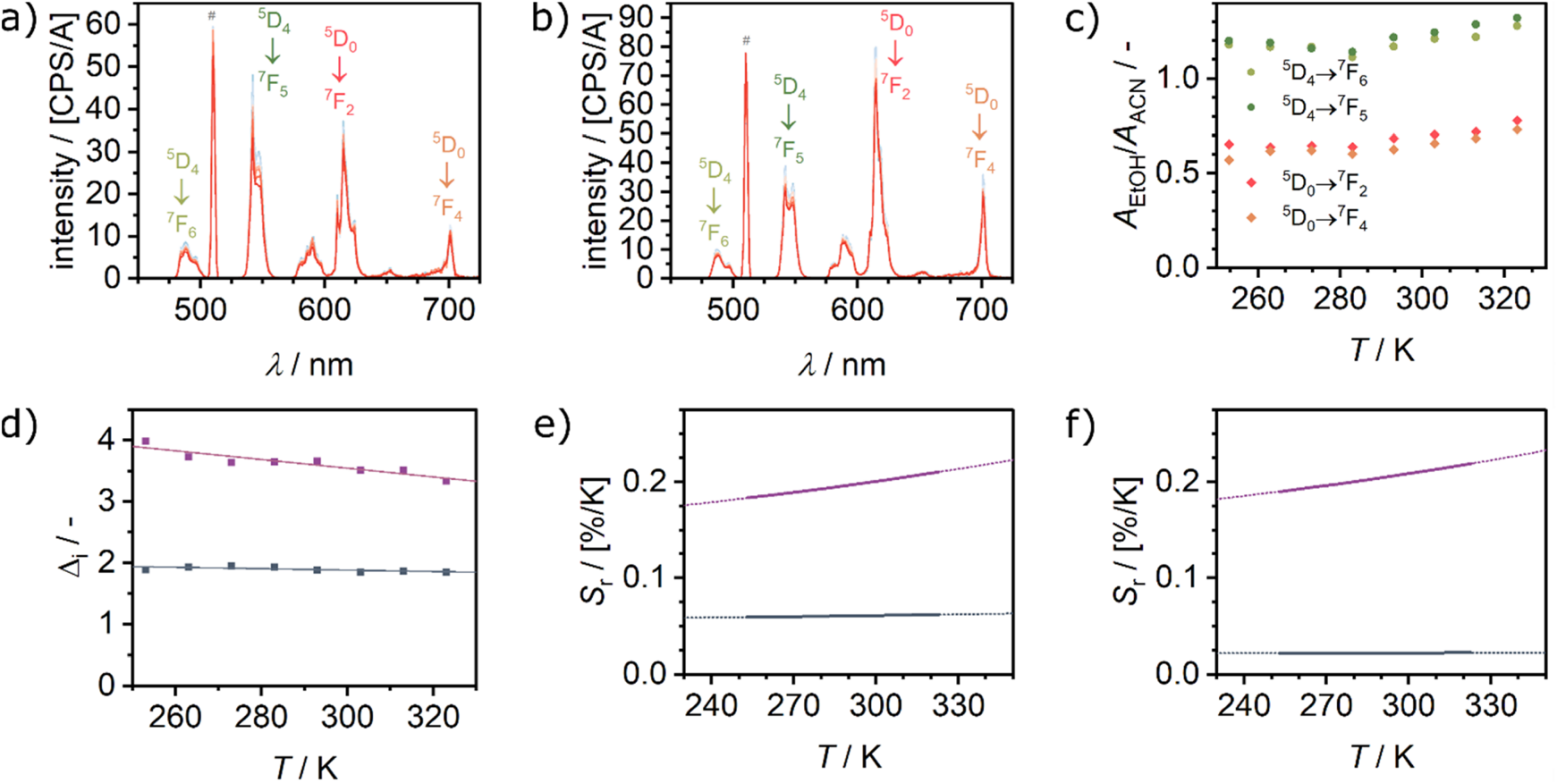
Temperature dependent luminescence spectra recorded between 253 and 333 K after excitation at 254 nm of Eu_0.02_Tb_0.98_BTC dispersed in (a) ethanol (EtOH) and (b) acetonitrile (ACN). # marks the 2^nd^ order laser peak. (c) Comparison of PL intensities of Tb^3+^ and Eu^3+^ transitions expressed as the ratio of the respective areas measured in EtOH and ACN. (d) Corresponding evolution of the luminescence ratios *Δ*_*i*_ as a function of temperature, for *Δ*_*i*_ based on Tb^3+ 5^D_4_ → ^7^F_5_ transition for dispersion in EtOH (purple) and ACN (blue). (e and f) *S*_r_ calculated using data for (e) Tb^3+ 5^D_4_ → ^7^F_5_ transition and (f) Tb^3+ 5^D_4_ → ^7^F_6_ transition. *S*_r_ of Eu_0.02_Tb_0.98_BTC dispersed in EtOH (purple) and ACN (blue). Solid lines correspond to *S*_r_ curves in the calibrated temperature regime, dotted lines correspond to the extrapolated *S*_r_ curves limited by the melting and boiling temperature of ACN and EtOH, respectively.

Eu_0.02_Tb_0.98_BTC was also investigated as temperature probe dispersed in ethanol (EtOH) or acetonitrile (ACN, Fig. 6) in the temperature range from 250 to 330 K. As for the pure MOFs (Fig. 3e and f), solvation with either ACN or EtOH tremendously affects the emission profile also for Eu_0.02_Tb_0.98_BTC. When dispersed in ACN, the Eu^3+ 5^D_0_ → ^7^F_2_ emission is stronger than the most intense Tb^3+^ emission (^5^D_4_ → ^7^F_5_), which might be explained by a better energy transfer from terbium to europium as compared to the dispersion in ethanol. The Tb^3+^ transitions are hardly affected by the nature of the solvent, with however a slightly lower intensity in ACN (Fig. 6c). Thus from *Δ*_*i*_ (eqn (1)) we can discern these solvents, as *Δ*_*i*_(EtOH) is approx. twice as high as *Δ*_*i*_(ACN), over the whole temperature range investigated (Fig. 6d and S16†). In contrast to the case in the solid state, where the Tb^3+^ transitions show a stronger thermal dependency than the Eu^3+^ transitions, in the dispersions both lanthanides are similarly affected. Therefore, we used a simplified linear expression to evaluate the temperature dependency of *Δ*_*i*_. The maximal sensitivity drops to 0.06% K^−1^ (^5^D_4_ → ^7^F_5_) and 0.22% K^−1^ (^5^D_4_ → ^7^F_5_) when dispersed in acetonitrile and ethanol, respectively. Interestingly, the only other report on the solvent effect on temperature sensing in MOFs by Cadiau *et al.* reported an inverse behaviour: when Tb_0.99_Eu_0.01_(bdc)_1.5_(H_2_O)_2_ was dispersed in water, the sensitivity increased from 0.14% K^−1^ (dry MOF) to 0.31% K^−1^.^61^

Here we note that the sensitivity is not affected by slight agglomeration and/or sedimentation which occur in the suspensions (Fig. S17 and S18†). The intensities of the different transitions of both lanthanides are similarly affected (Fig. S17a†) due to their homogeneous incorporation within a particle (see above). Thus, the luminescence ratios *Δ*_*i*_ as a descriptor for the temperature sensing are not affected over time (Figure S17b†).

In summary, from *Δ*_*i*_ the solvents can readily be discerned, while temperature determination is possible using the temperature dependency of *Δ* and of the sensitivity *S*_r_.

## Conclusions

Using the acetate mediated synthesis protocol of LnBTCs, we obtained a series of Eu_*x*_Tb_(1−*x*)_BTC MOFs, in which the ratio of the two lanthanides is controlled by the synthesis gel composition. Using ^1^H MAS NMR spectroscopy, we demonstrated the homogeneous incorporation of both cations in the series of mixed metal Eu_*x*_Tb_(1−*x*)_BTC MOFs. The ^1^H NMR chemical shifts of the two crystallographically different protons change continuously in the series from one pure LnBTC MOF to the other, indicating the formation of a single crystalline phase with homogeneous distribution of the two different metals. The precise control of lanthanide stoichiometry and distribution allows to control the luminescent properties of the final material. While for Eu loadings above 25 mol%, the Tb^3+^ emission is completely quenched, in Eu_0.05_Tb_0.95_BTC still 20% of the Tb^3+^ emission intensity of the parent TbBTC remains, sufficient to be used as temperature sensor. Eu_0.05_Tb_0.95_BTC and Eu_0.02_Tb_0.98_BTC allow for ratiometric temperature sensing in the range between 200 and 330 K with unprecedented sensitivities for pure Tb–Eu MOFs. Interestingly, the emission profiles of Tb centres are hardly affected when the material is dispersed in different solvents, while for Eu centres the emission wavelengths of the transitions as well as the intensity ratios for the different transitions change in the presence of ethanol and acetonitrile. Therefore, the solvation of the MOFs should be considered when using them as ratiometric temperature sensors in the presence of solvents or vapours. This solvation dependency of the lanthanide emission can be rationalised and taken into account in such sensors, to determine temperature and discern between different solvents present as demonstrated here for acetonitrile and ethanol as model compounds.

## Experimental

### Materials and methods

Eu(NO_3_)_3_·6H_2_O (Thermo Scientific, 99.9%), Tb(NO_3_)_3_·6H_2_O (Thermo Scientific, 99.99%), 1,3,5-benzene tricarboxylic acid (H_3_BTC, Alfa Aesar, 98%), *N*,*N*-dimethylformamide (DMF, Merck, 99.5%), NaOAc (Merck, 99%) and ethanol (Merck, >95%). All chemicals were used as supplied without further purification.

Powder X-ray diffraction (PXRD) patterns were collected on a PANalytical Emryrean series 2 in Bragg–Brentano geometry using CuKα radiation at 2*θ* angles between 2 and 50°. Thermogravimetric analysis (TGA) of powder samples was measured on a Hi-Res TGA 2950 Thermogravimetric Analyzer (TA Instruments) in the temperature range from room temperature to 700 °C with a heating rate of 2 K min^−1^ in synthetic air and on a SDT 2960 Simultaneous DSC-TGA (TA Instruments) in the temperature range from room temperature to 1100 °C with a heating rate of 2 K min^−1^ in synthetic air. Nitrogen physisorption experiments were carried out using an ASAP 2000 (Micromeritics). Prior to measurements the samples were degassed at 250 °C under vacuum overnight. Inductively coupled plasma optical emission spectroscopy analysis (ICP OES) was done using a SPECTRO Ciros-CCD. Prior to ICP OES analysis, ∼100 mg of MOF powder were digested in aqua regia (9 ml HCl, 3 ml HNO_3_) inside a Teflon lined autoclave using a Berghof-Microwave Digester-Speedwave 4 (*θ*_max_ = 200 °C, *p*_max_ = 50 bar, 37 min). IR spectra were recorded using a JASCO FT/IR-4100 in attenuated total reflectance geometry (ATR, resolution 2 cm^−1^, 64 scans).

Photoluminescence (PL) spectra of the suspensions were recorded using either a fluorescence spectrophotometer (Horiba Fluorolog) in the temperature range of −253 to 323 K or using a Jasco Spectrofluorometer FP-8500 at room temperature. PL spectra at room temperature of the solid samples were recorded using a Jasco Spectrofluorometer FP-8500 (*λ*_ex_ = 256 nm).

Steady-state PL spectra in the low temperature regime (200–300 K) were recorded with a fluorescence spectrophotometer Fluorolog-3 (FL3-22) by Horiba Scientific with a xenon lamp operating at 370 nm as excitation source. Time resolved PL decays were recorded with a phosphorimeter (FL-1040A) integrated into the Fluorolog-3 setup with a pulsed xenon lamp operating at 370 nm as excitation source. Temperature adjustment between 200 and 330 K was achieved by using a JANIS ST-500 cryostat. Sample preparation for the temperature dependent PL spectroscopy (steady-state and time resolved) of the solid samples was done by dispersing the MOF powder in ethanol and drop casting 70 μl onto a silicon substrate.

Time correlated single photon counting (TCSPC) data were recorded using a phosphorimeter (FL-1040A) integrated into the Fluorolog-3 setup with a pulsed xenon lamp operating at 370 nm as excitation source. Temperature adjustment between 200 and 320 K was achieved by using a modified ST-300-MS-cryostat (Janis Research). The values reported in Table S2† are the amplitude average lifetime from deconvolution of the TCSPC data using:5
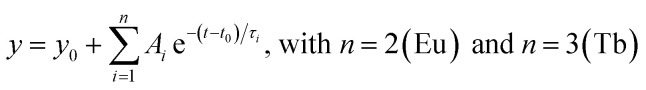


In case of the Eu emission in Eu_0.02_Tb_0.98_BTC materials, also the build-up time constant was integrated into the formula used for deconvolution:6
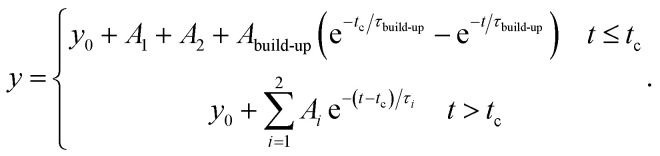


Deconvolution was done using Origin 2023.

Steady-state PL spectra in the high temperature regime (298–473 K) as well as the full temperature regime (173–473 K) were recorded with a home-build fibre optical fluorescence spectrophotometer consisting of an AvaSpec-HSC1024x58TEC-EVO spectrometer (Avantes) and a 385 nm LED (Thorlabs) as excitation source. Spectra were recorded in reflectance. Temperature adjustment between 173 K and 473 K was achieved by using a FTIRSP600 stage (Linkam Scientific Instruments). Solid samples were deposited as powders on quartz lids on top of the silver heating block inside the FTIRSP600 stage.

Scanning electron microscopy (SEM) images were obtained on a Carl Zeiss Gemini Ultra 55. Dynamic scanning calorimetry was recorded using a NETZSCH DSC 204F1 Phoenix equipped with a liquid nitrogen dewar. Samples were filled into aluminum pans, closed with an aluminum lid and analysed between −30 and 100 °C with a heating/cooling rate of 2 K min^−1^.

^1^H solid state NMR spectra were collected on a Varian 500 NMR spectrometer (^1^H resonance frequency at 499.86 MHz) equipped with a 1.6 mm probe at a spinning frequency of 30 kHz. Prior to NMR measurements, all samples were activated overnight at 250 °C under vacuum and packed in an Argon filled glovebox. The DEPTH sequence was used for recording all spectra. The π/2 and π pulse widths for proton were 2.5 μs and 5.0 μs, respectively. The recycle delay was 50 ms (*T*_1_ ≈ 9 ms) with an acquisition time of 3 ms. 1024 scans were accumulated. All ^1^H NMR spectra were referenced to 0.0 ppm to the central line of sodium trimethylsilylpropanesulfonate.

### Synthetic procedures

General procedure for synthesis of nanorod Eu_*x*_Tb_(1−*x*)_BTC MOFs. The MOFs were synthesised following a modified literature procedure.^25^ In a typical synthesis of mixed LnBTC (Eu_0.5_Tb_0.5_BTC), 373.4 mg (4.5 mmol) NaOAc were dissolved in 57 ml DMF and 3 ml deionised H_2_O in a 100 ml round bottom flask equipped with a reflux condenser. Next, 339.8 mg (750 μmol) Tb(NO_3_)_3_·6H_2_O, 334.6 mg (750 μmol) Eu(NO_3_)_3_·6H_2_O and 321.7 mg (1.5 mmol) H_3_BTC were added and the synthesis mixture was heated to 110 °C and stirred at this temperature for 4 h (300 rpm). After cooling to room temperature, the white solid was isolated by centrifugation, washed with *ca.* 30 ml DMF and 30 ml absolute ethanol (three times). The white slurry was dried in an oven at 80 °C. Isolated yield: 471.3 mg (1.02 mmol, 68%).

All Eu_*x*_Tb_(1−*x*)_BTC samples were prepared following the procedure described above and are denoted accordingly to the experimentally determined molar ratios as: Eu_*x*_Tb_(1−*x*)_BTC MOFs [*x* = 0 to 1].

## Data availability

Data for this article, including information on the data sets used in Fig. 1–6 (raw data and data converted to Ascii or JCAMP files) and information on data processing, are available at https://doi.org/10.5281/zenodo.14773089.

## Author contributions

M. J.: investigation (MOF synthesis), writing – review & editing. M. R.: investigation (TD-PL solid-state, TCSPC), writing – review & editing. Z. W.: investigation (TD-PL dispersion), writing – review & editing. S. M.: investigation (MOF synthesis, TD-PL solid-state), formal analysis, writing – review & editing. R. N., C. H.: investigation (solid-state NMR), formal analysis, writing – review & editing. R. E. R.: investigation (additional characterisation), writing – review & editing. M. F.: investigation (additional characterisation), supervision, writing – review & editing. R. F., F. M. W.: conceptualization, supervision, formal analysis, writing – original draft, review & editing. K. M.: writing – review & editing. D. W.: conceptualization, supervision, writing – original draft, review & editing, funding acquisition. D. S., G. B., M. H.: conceptualization, supervision, writing – review & editing, funding acquisition.

## Conflicts of interest

There are no conflicts to declare.

## Supplementary Material

RA-015-D5RA00822K-s001
