# Supplementary material for: Europium(iii)/terbium(iii) mixed metal–organic frameworks and their application as ratiometric thermometers with tuneable sensitivity in organic dispersion
Source: RSC Adv. 2025 Apr 9;15(14):11230–42. doi: 10.1039/d5ra00822k (PMC11979743; doi:10.1039/d5ra00822k)
Supplement: RA-015-D5RA00822K-s001 [file RA-015-D5RA00822K-s001.pdf]

Supplementary Information (SI) for RSC Advances.
This journal is © The Royal Society of Chemistry 2025

Supporting Information

Europium(III)/Terbium(III) Mixed Metal-Organic Frameworks and their Application as Ratiometric Thermometers with Tuneable Sensitivity in Organic Dispersion

Madhura Joshi, Maurizio Riesner, Zhuang Wang, Sahba Mireskandari, Raju Nanda, Rebecca Reber, Christoph Huber, Marcus Fischer, Rachel Fainblat, Karl Mandel, Dorothea Wisser, Doris Segets, Gerd Bacher, Florian M. Wisser, and Martin Hartmann

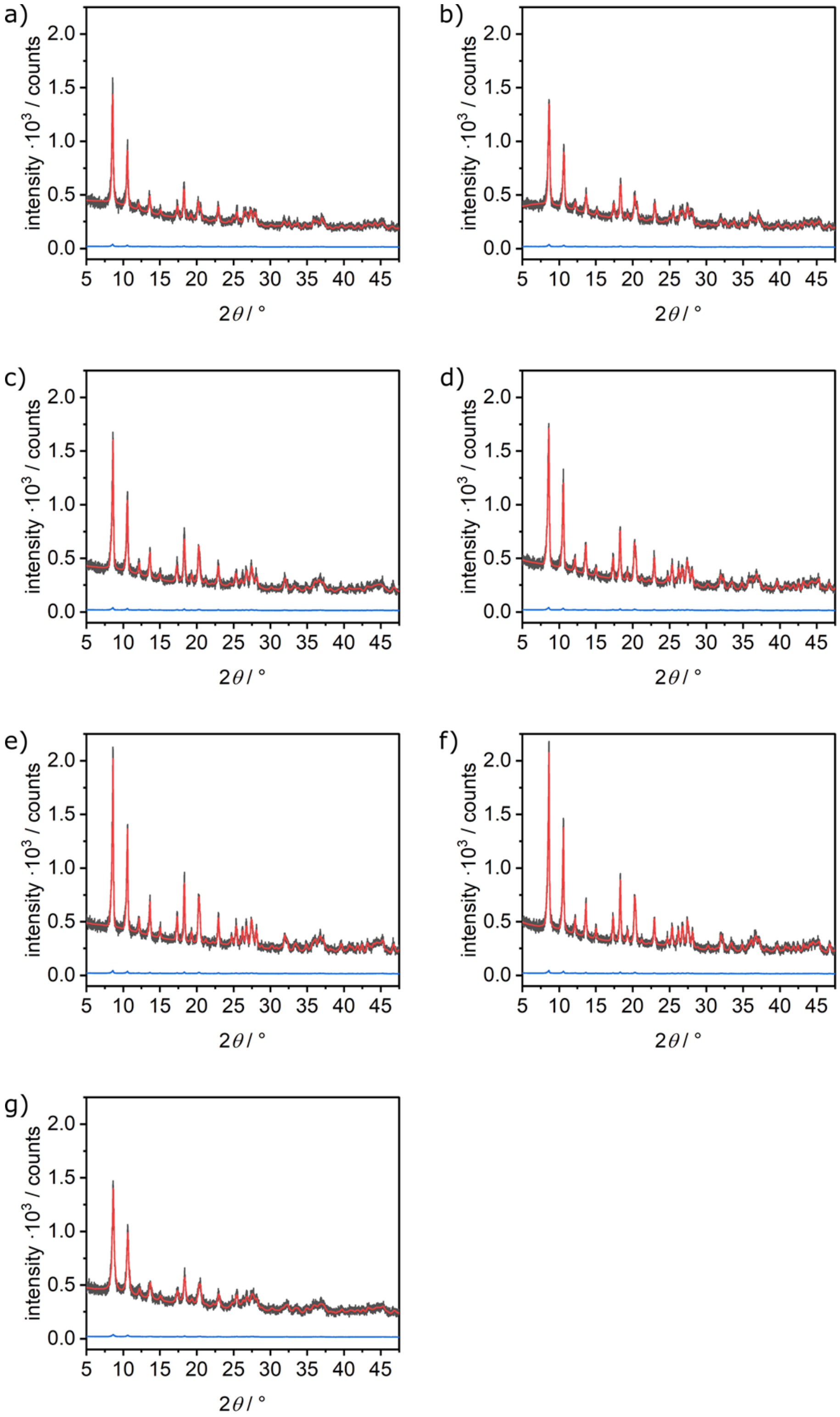

Figure S1. Le Bail refinement of a) EuBTC (GOF = 1.30, Rp = 5.80, wRp = 7.37), b) $Eu_{0.75}Tb_{0.25}BTC$ (GOF = 1.25, Rp = 5.59, wRp = 7.13), c) $Eu_{0.51}Tb_{0.49}BTC$ (GOF = 1.42, Rp = 6.25, wRp = 8.00), d) $Eu_{0.25}Tb_{0.75}BTC$ (GOF = 1.12, Rp = 4.71, wRp = 6.01), e) $Eu_{0.05}Tb_{0.95}BTC$ (GOF = 1.14, Rp = 4.66, wRp = 5.99), f) $Eu_{0.02}Tb_{0.98}BTC$ (GOF = 1.18, Rp = 4.88, wRp = 6.20) and g) TbBTC (GOF = 1.18, Rp = 4.82, wRp = 6.18). Le Bail refinement was performed using Jana 2020.[1]

Table S1. Overview of estimated crystallite size *L* by averaging the crystallite size obtained using the Scherer equation for the first four peaks. Please note that these values represent a rough estimation due to the asymmetric peak shape and the unknown form factor (assumed to be 0.9).

Material	EuBTC	$Eu_{0.75}Tb_{0.25}BTC$	$Eu_{0.51}Tb_{0.49}BTC$	$Eu_{0.25}Tb_{0.75}BTC$	$Eu_{0.05}Tb_{0.95}BTC$	$Eu_{0.02}Tb_{0.98}BTC$	TbBTC
L / nm	38.0 ± 5.3	35.1 ± 2.4	37.1 ± 2.6	35.7 ± 5.3	44.6 ± 2.5	46.4 ± 2.7	27.9 ± 5.7

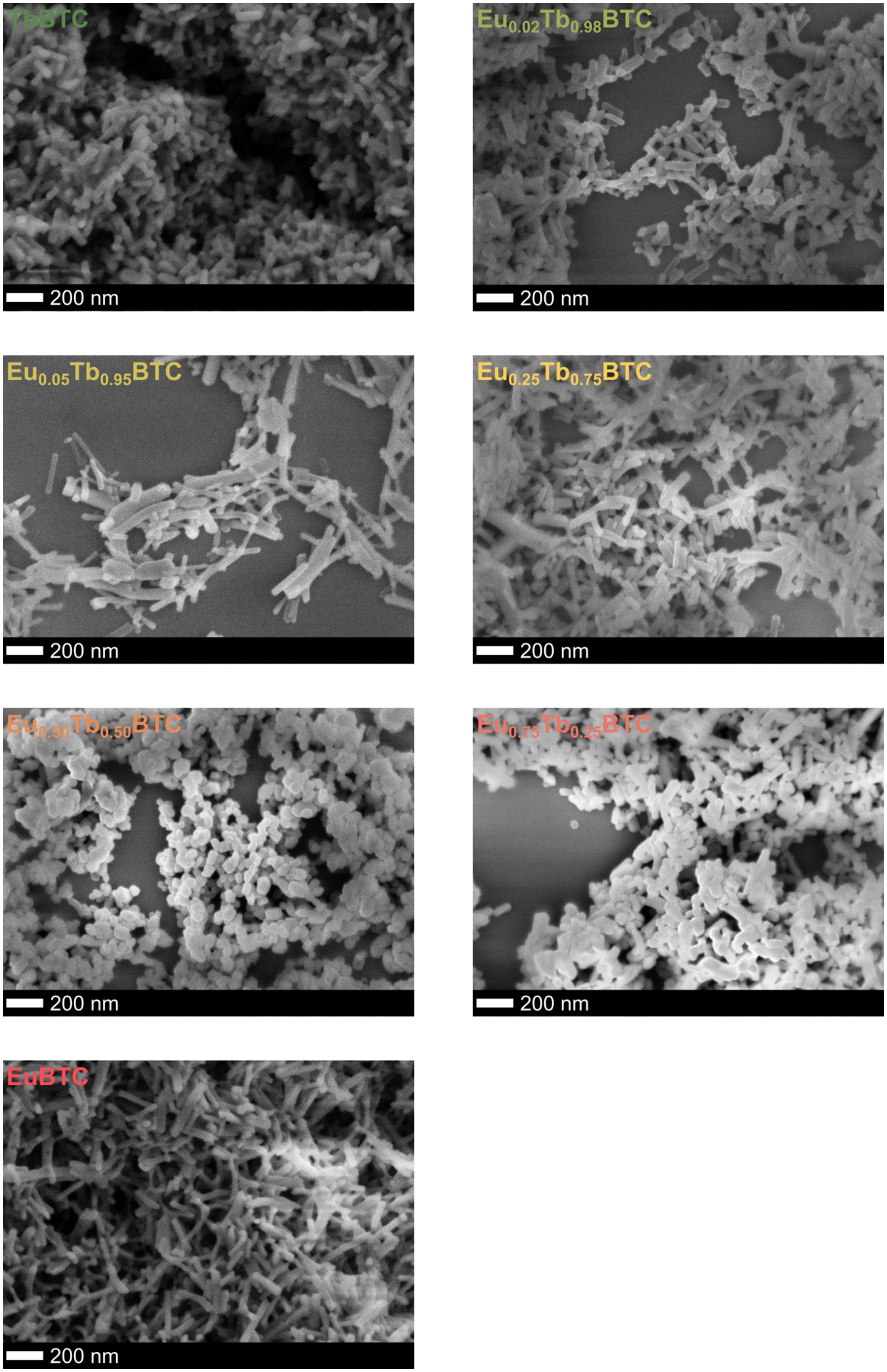

Figure S2. SEM images of the series of $Eu_xTb_{1-x}BTC$ MOFs highlighting the characteristic shape of the particles. Scale bar 200 nm.

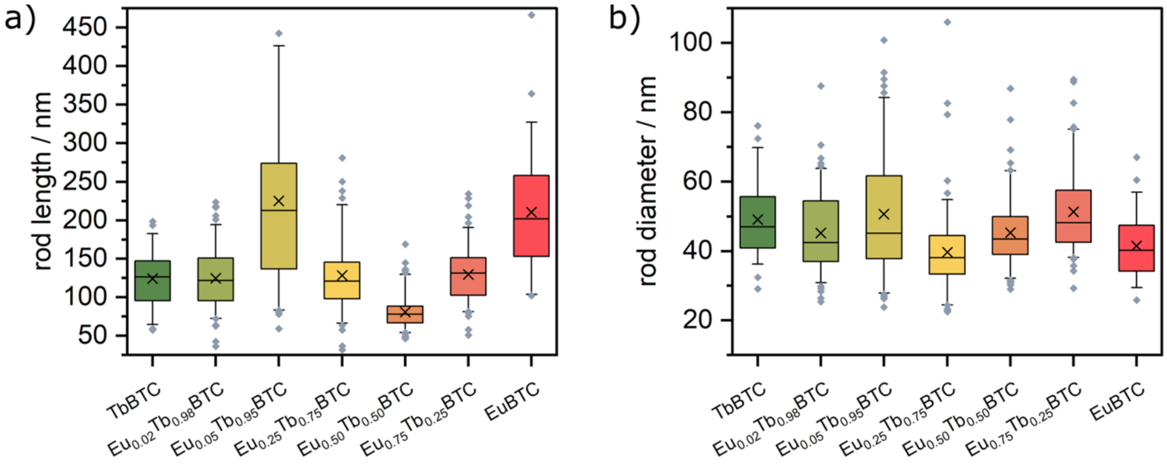

Figure S3. Box diagrams of rod length and rod width diameter for the series of $Eu_xTb_{1-x}BTC$ MOFs. The box diagram shows the upper and lower quartiles (box), the mean (cross) and the median of the distribution (line) together with the 5th and 95th percentiles (whisker) and outliers (grey diamonds). For each dataset between 50 and 100 individual particles from different images were evaluated using ImageJ.

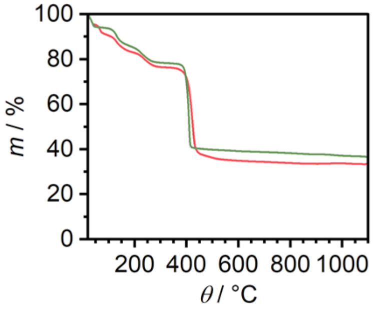

Figure S4. Additional TGA recorded under synthetic air (2K/min heating rate) from RT to 1100°C of EuBTC (red) and TbBTC (green).

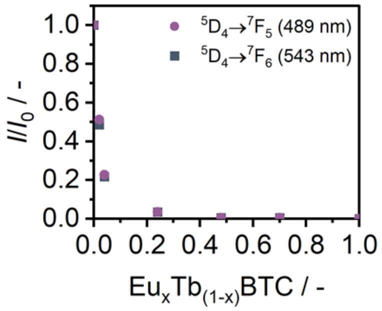

Figure S5. Quenching ratio of the Tb emission at 489 nm (purple) and 543 nm (dark grey) as a function of MOF composition.

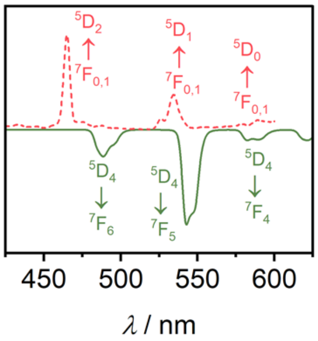

Figure S6. Comparison of EuBTC excitation spectrum (red dotted) and TbBTC PL spectrum (green).

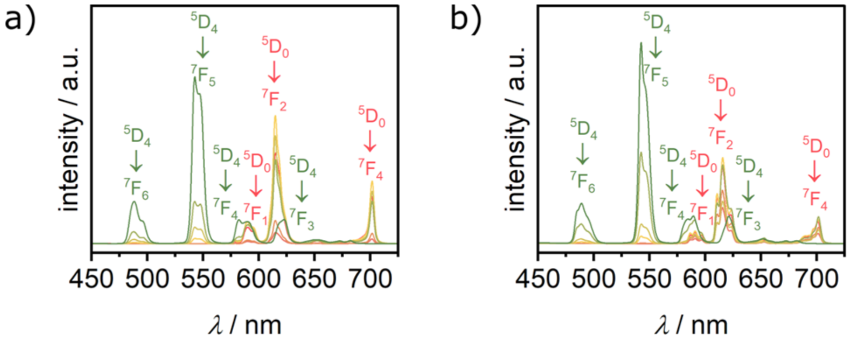

Figure S7. PL spectra of the $Eu_xTb_{(1-x)}BTC$ series dispersed in a) acetonitrile and b) ethanol. Approx. 1 mg MOF was dispersed in 10 ml solvent.

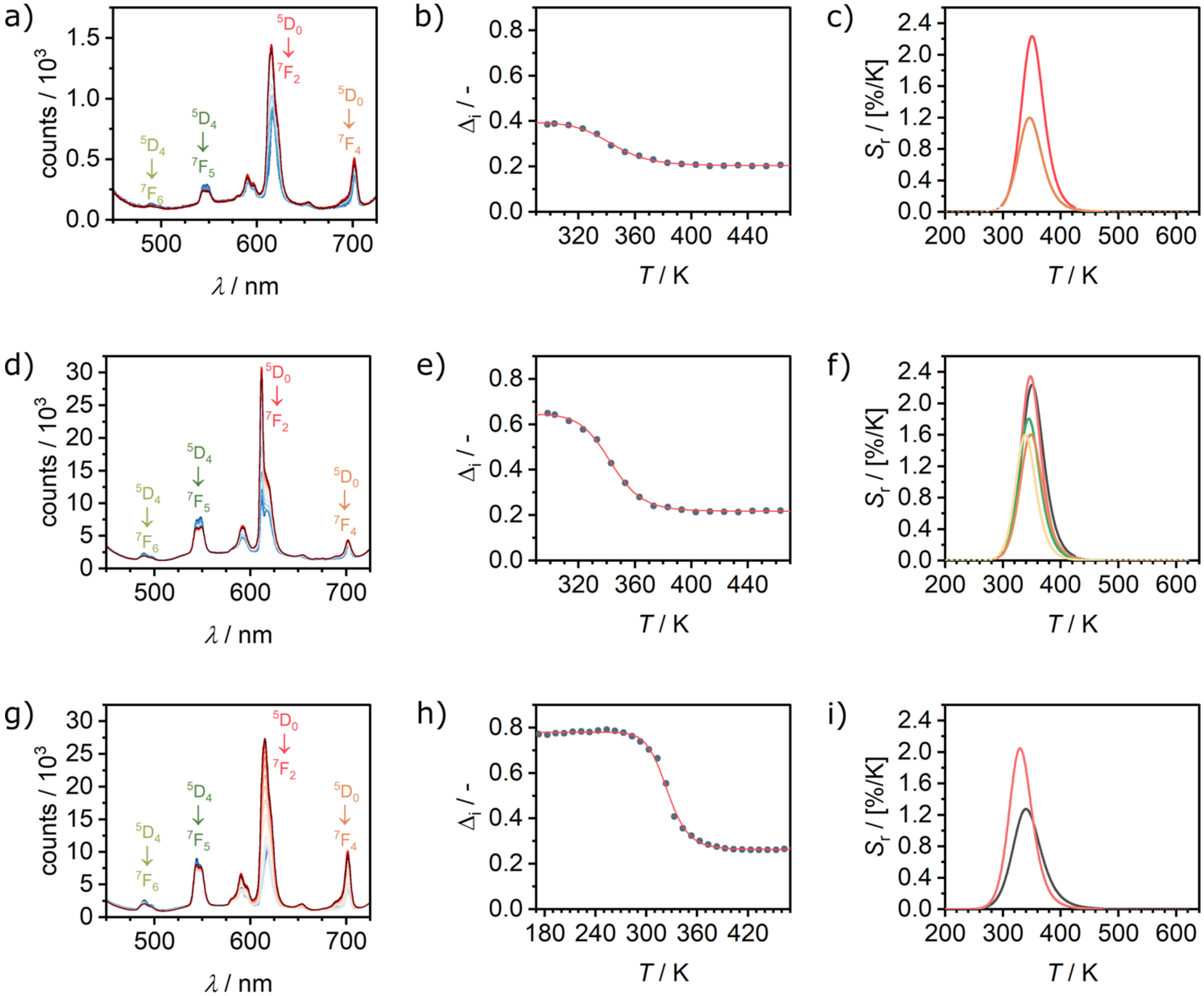

Figure S8. Temperature dependent luminescence spectra recoded between 293 and 473 K after excitation at 385 nm of $Eu_{0.05}Tb_{0.95}BTC$ (a) and $Eu_{0.02}Tb_{0.98}BTC$ (d). Evolution of the luminescence ratios Δ_i as a function of temperature, for $Eu_{0.05}Tb_{0.95}BTC$ (b) and b) $Eu_{0.02}Tb_{0.98}BTC$ (e). Evolution of relative sensitivity in the analysed temperature regime for Tb $^5D_4 \rightarrow ^7F_5$ transition. (c) S_r of $Eu_{0.02}Tb_{0.98}BTC$ (red), S_r of $Eu_{0.05}Tb_{0.95}BTC$ (orange). Solid lines correspond to S_r curves in the calibrated temperature regime, dotted lines correspond to the extrapolated S_r curves limited by the thermal decomposition of the MOFs around 623 K (Figure 1b). (f) Comparison of S_r of $Eu_{0.02}Tb_{0.98}BTC$ for five independent experiments. (g) Temperature dependent luminescence spectra recoded between 173 and 473 K after excitation at 385 nm of $Eu_{0.02}Tb_{0.98}BTC$. (h) Corresponding evolution of the luminescence ratios Δ_i as a function of temperature. Evolution of relative sensitivity in the analysed temperature regime for Tb $^5D_4 \rightarrow ^7F_5$ transition. (i) Comparison of S_r of $Eu_{0.02}Tb_{0.98}BTC$ for two independent measurements. Solid lines correspond to S_r curves in the calibrated temperature regime, dotted lines correspond to the extrapolated S_r curves limited by the thermal decomposition of the MOFs around 623 K (Figure 1b).

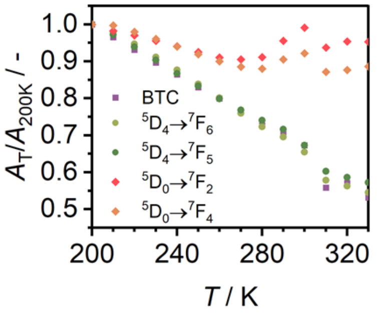

Figure S9. Evolution of the emission with temperature, expressed as area at a given temperature divided by the area at 200 K, for $Eu_{0.05}Tb_{0.95}BTC$. The jump at 300 K is an instrumental artefact.

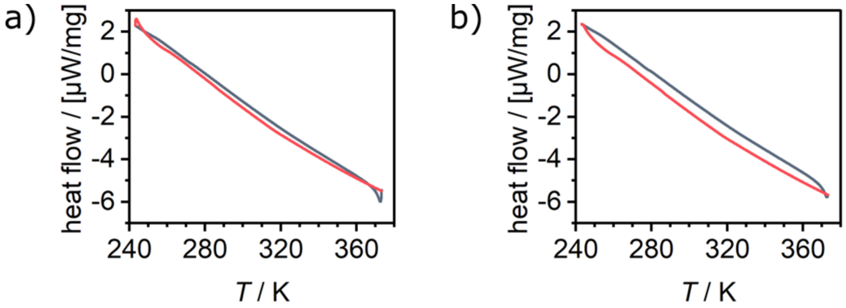

Figure S10. DSC curves of a) $Eu_{0.02}Tb_{0.98}BTC$ and b) $Eu_{0.05}Tb_{0.95}BTC$ recorded with a cooling/heating rate of 2K/min.

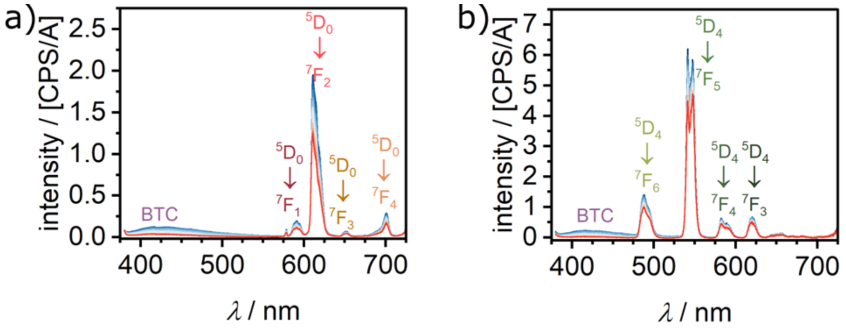

Figure S11. Temperature dependent luminescence spectra recoded between 200 and 330 K after excitation at 370 nm of a) EuBTC and b) TbBTC.

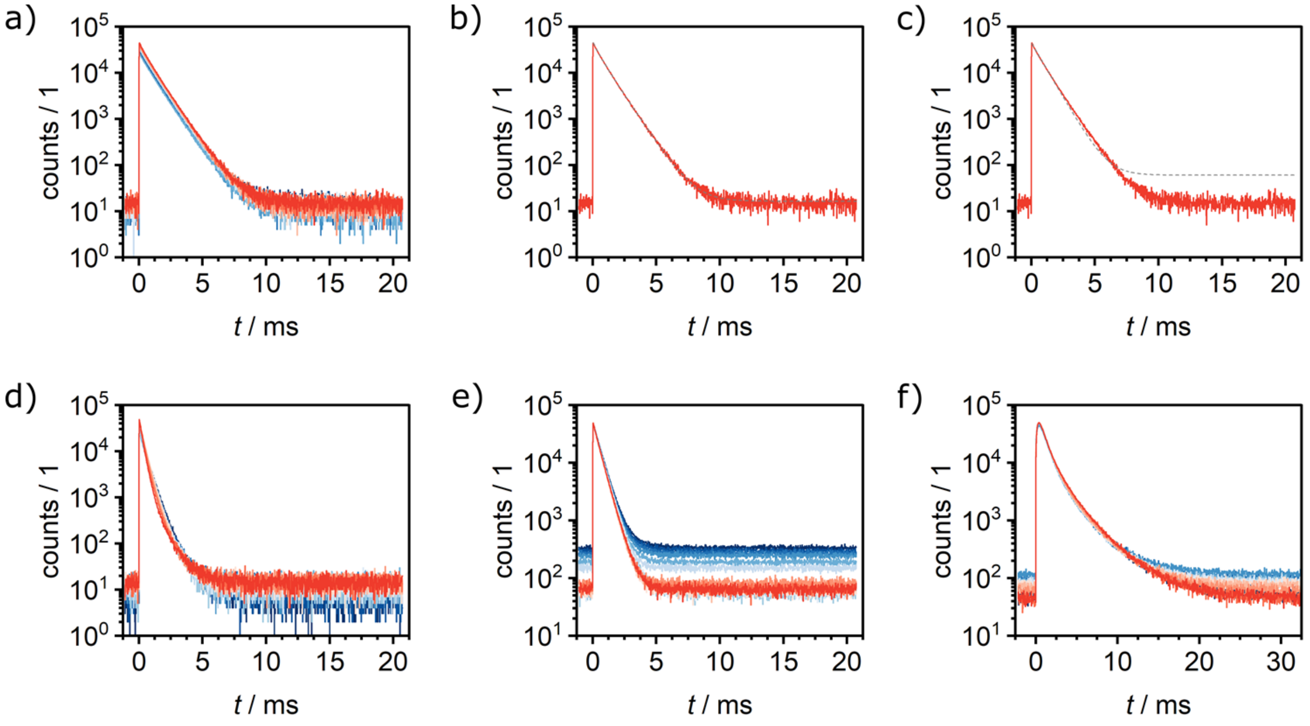

Figure S12. a) Time correlated single photon counting (TCSPC) spectra of TbBTC measured in steps of 10 K between 200 K (dark blue) and 320K (red) recorded at λ_{em} = 542 nm (Tb emission). b, c) Comparison of deconvolution of the TCSPC spectra at 320K (shown in a) using a sum of three exponential functions (b, *n*=3 in equation 5, main text) and using only one exponential function (c, *n*=1 in equation 5, main text). d) TCSPC spectra of $Eu_{0.02}Tb_{0.98}BTC$ measured in steps of 10 K between 200 K (dark blue) and 320K (red) recorded at λ_{em} = 542 nm (Tb emission). e) TCSPC spectra of EuBTC and f) of $Eu_{0.02}Tb_{0.98}BTC$ measured in steps of 10 K between 200 K (dark blue) and 320K (red) recorded at λ_{em} = 610 nm (Eu emission).

Table S2. Overview of luminescence lifetime for Tb (τ_{Tb}) and Eu (τ_{Eu}) centered emissions together with buildup time constant ($\tau_{buildup}$) of the Eu emission in $Eu_{0.02}Tb_{0.98}BTC$ and efficiency of the energy transfer. [a] largest error applied form two independent measurements of $Eu_{0.02}Tb_{0.98}BTC$.

	TbBTC [a]	EuBTC [a]	$Eu_{0.02}Tb_{0.98}BTC$			
T /K	τ_{Tb} / µs	τ_{Eu} / µs	τ_{Tb} / µs	η_{ET} / %	τ_{Eu} / µs	$\tau_{build-up}$ / µs
200	979 ± 66	546 ± 37	422 ± 29	56.9 ± 4.2	1355 ± 46	116 ± 22
210	979 ± 66	542 ± 36	415 ± 29	54.6 ± 4.2	1340 ± 48	116 ± 22
220	978 ± 66	531 ± 36	404 ± 31	58.6 ± 4.5	1329 ± 54	116 ± 21
230	939 ± 63	527 ± 35	410 ± 21	56.4 ± 3.2	1317 ± 55	117 ± 21
240	951 ± 64	521 ± 35	392 ± 15	58.7 ± 2.3	1313 ± 56	120 ± 19
250	944 ± 63	541 ± 36	393 ± 22	58.4 ± 3.3	1298 ± 55	120 ± 17
260	942 ± 63	505 ± 34	388 ± 21	58.8 ± 3.2	1289 ± 61	122 ± 16
270	938 ± 63	524 ± 35	382 ± 14	59.3 ± 2.1	1288 ± 55	126 ± 14
280	941 ± 63	499 ± 33	369 ± 11	60.8 ± 1.6	1296 ± 36	124 ± 13
290	943 ± 63	495 ± 33	342 ± 10	63.7 ± 1.5	1291 ± 53	133 ± 11
300	925 ± 63	498 ± 33	310 ± 9	66.4 ± 1.3	1304 ± 46	138 ± 10
310	921 ± 62	499 ± 33	309 ± 3	66.5 ± 0.5	1329 ± 23	144 ± 6
320	902 ± 60	505 ± 34	274 ± 5	69.6 ± 0.8	1332 ± 23	188 ± 27

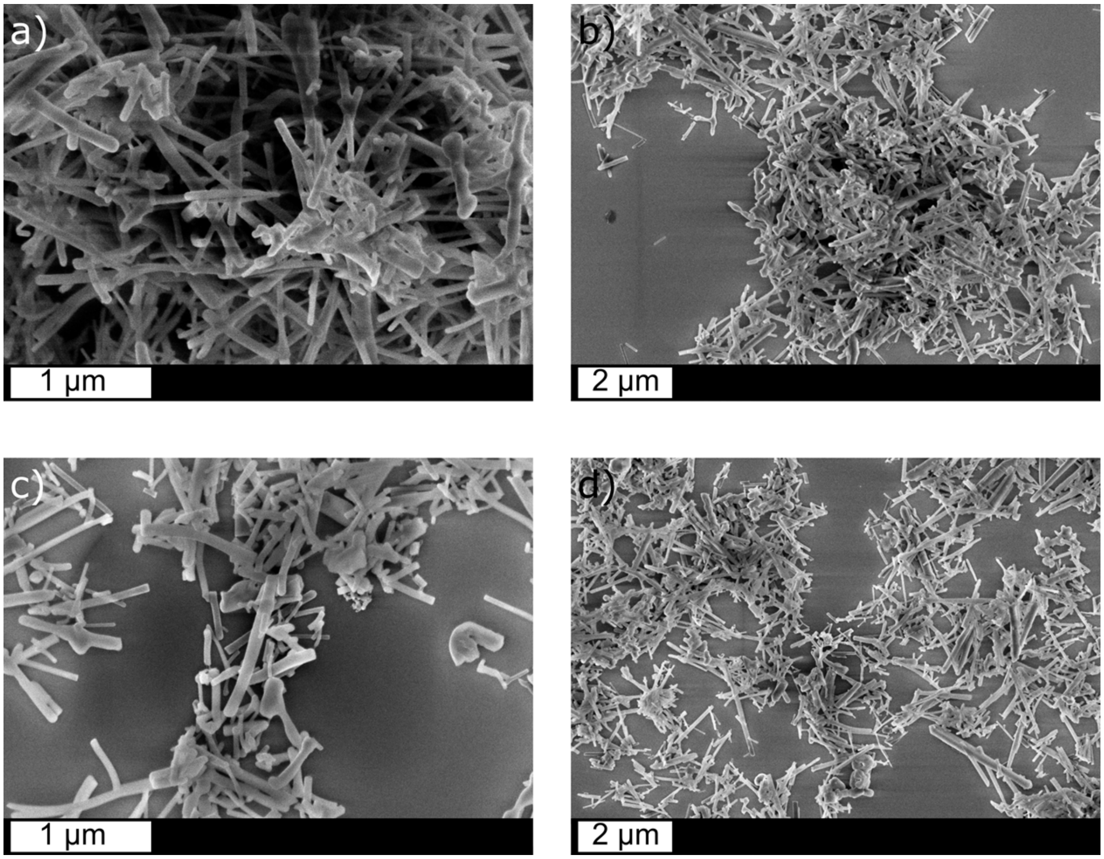

Figure S13. SEM images of $Eu_{0.02}Tb_{0.98}BTC$ before (a, b) and after (c, d) ratiometric temperature sensing. Materials were re-dispersed in ethanol by ultrasonication and drop-coated onto silicon wavers prior to SEM analysis.

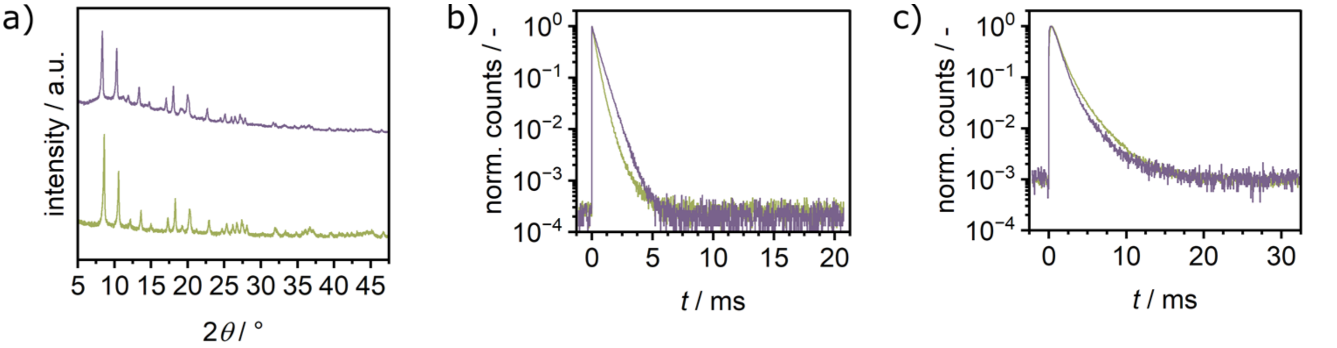

Figure S14. a) PXRD patterns of as-synthesized $Eu_{0.02}Tb_{0.98}BTC$ (green) and spent $Eu_{0.02}Tb_{0.98}BTC$ (purple) after ratiometric temperature sensing. The estimated crystallite sizes, as obtained using the Scherrer equation, is statistically the same with values of 46±3 nm (before sensing) and 42±2 nm (after sensing). b), c) Comparison of Tb (b) and Eu (c) emission lifetime of as-synthesized $Eu_{0.02}Tb_{0.98}BTC$ (green) and spent $Eu_{0.02}Tb_{0.98}BTC$ (purple) after ratiometric temperature sensing. τ_{Tb} changes from 336 ± 23 µs (pristine) to 493 ± 35 µs (spent), while τ_{Eu} varies within the error from 1123 ± 79 µs (pristine) to 1021 ± 71 µs (spent) $Eu_{0.02}Tb_{0.98}BTC$. As no structural changes are observed, those changes are most likely due to desorption of remaining guest molecules.

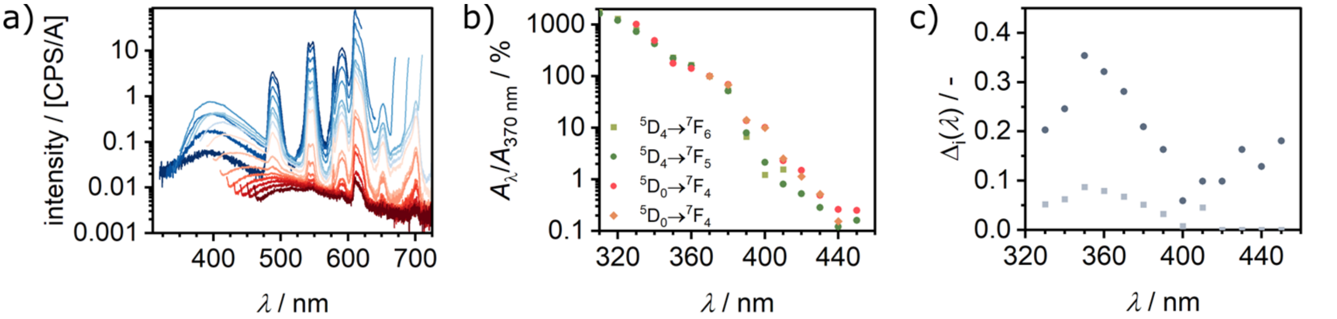

Figure S15. a) PL spectra of $Eu_{0.05}Tb_{0.95}$BTC b) evolution of the relative area of the main Tb and Eu emission bands and c) evolution of the luminescence ratios Δ_i as a function of excitation wavelength (from λ_{ex} = 310 nm (dark blue) to λ_{ex} = 450 nm (dark red)). Data for Δ_i based on Tb $^5D_4 \rightarrow {}^7F_5$ transition are shown in dark grey, based on Tb $^5D_4 \rightarrow {}^7F_6$ transition in light grey.

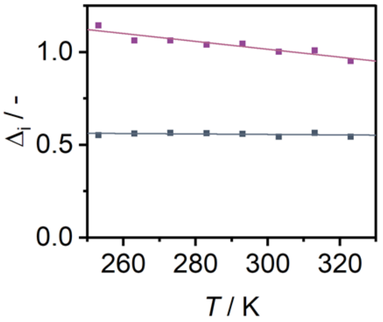

Figure S16. Evolution of the luminescence ratios Δ_i as a function of temperature, for Δ_i based on Tb^{3+} $^5D_4 \rightarrow {}^7F_6$ transition for dispersion in EtOH (purple) and ACN (blue).

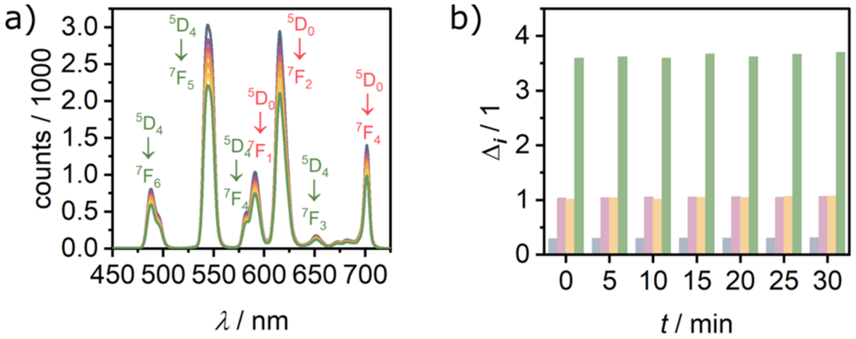

Figure S17 a) Evolution of the luminescence of an ethanolic dispersion of $Eu_{0.02}Tb_{0.98}$BTC over 30 minutes (interval of 5 minutes). The dispersion was directly prepared after the last washing step of the synthesis, thus avoiding drying and redispersion of the nanorods. b) Evolution of the luminescence ratios Δ_i as a function of time for $\Delta_i = \frac{A_{Tb(^5D_4 \rightarrow ^7F_6)}}{A_{Eu(^5D_0 \rightarrow ^7F_2)}}$ (blue), $\Delta_i = \frac{A_{Tb(^5D_4 \rightarrow ^7F_5)}}{A_{Eu(^5D_0 \rightarrow ^7F_2)}}$ (red), $\Delta_i = \frac{A_{Tb(^5D_4 \rightarrow ^7F_6)}}{A_{Eu(^5D_0 \rightarrow ^7F_4)}}$ (yellow) and $\Delta_i = \frac{A_{Tb(^5D_4 \rightarrow ^7F_5)}}{A_{Eu(^5D_0 \rightarrow ^7F_4)}}$ (green), demonstrating the stability of the luminescence ratios albeit slightly agglomeration and/or sedimentation of the MOF particles (see a) and **Figure S15**, **Table S3**).

Table S3. Overview of agglomerate diameter, agglomerate contribution (area) and average hydrodynamic radius of suspension in EtOH for $Eu_{0.02}Tb_{0.98}$BTC. The suspension was prepared form dried particles. At each time (0, 2.5 and 4 h) three runs were performed and the average data are shown. For each run, the measurement position as well as the number of runs were automatically re-optimized to achieve stable signals. [a] only seen in one out of three experiments.

Solvent	t / h	d_1 / µm	A_1 / %	d_2 / µm	A_2 / %	d_3 / µm	A_3 / %	r_H / µm
ethanol	0	0.48 ± 0.03	21 ± 1	4.1 ± 0.2	79 ± 1			1.58 ± 0.04
	2.5	0.31 ± 0.11	12 ± 4	4.6 ± 1.2	88 ± 4			3.06 ± 0.56
	4			2.04 ± 0.06	95 ± 4	7.17 [a]	5 ± 4 [a]	3.18 ± 0.02

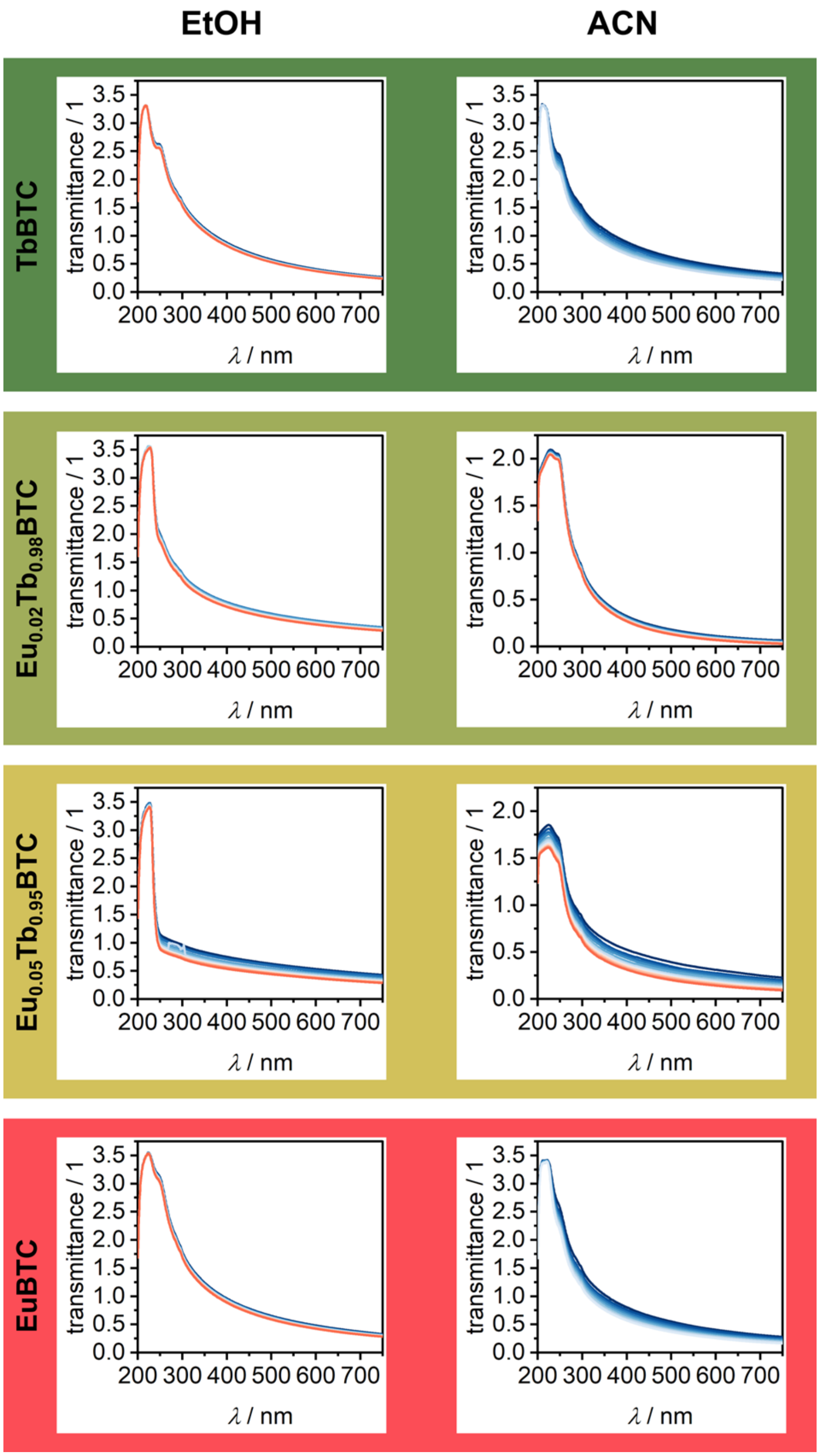

Figure S18. Evolution of transmittance of ethanolic dispersions of $Eu_{0.05}Tb_{0.95}BTC$ (a) and $Eu_{0.02}Tb_{0.98}BTC$ (b). The dispersions were directly prepared after the last washing step of the synthesis, thus avoiding drying and redispersion of the nanorods. Measurements were performed from t = 0 min (blue) to t = 60 min (red) with increments of 5 minutes.

References

1 V. Petříček, L. Palatinus, J. Plášil and M. Dušek, Jana2020 – a new version of the crystallographic computing system Jana, *Z. Kristallogr. - Cryst. Mater.*, 2023, **0**. DOI: 10.1515/zkri-2023-0005.
